# Antibacterial effectors in *Dictyostelium discoideum*: specific activity against different bacterial species

**DOI:** 10.1128/msphere.00471-24

**Published:** 2024-10-08

**Authors:** Raphael Munoz-Ruiz, Otmane Lamrabet, Tania Jauslin, Cyril Guilhen, Alixia Bourbon, Pierre Cosson

**Affiliations:** 1 Department of Cell Physiology and Metabolism, Faculty of Medicine, University of Geneva, Geneva, Switzerland; University of Nebraska Medical Center College of Medicine, Omaha, Nebraska, USA

**Keywords:** *Dictyostelium discoideum*, Gram-negative bacteria, Gram-positive bacteria, bacteriolytic proteins, sulfated proteins, glycosylation, Kil1, ModA

## Abstract

*Dictyostelium discoideum* is a phagocytic amoeba continuously eating, killing, and digesting bacteria. Previous studies have detected in *D. discoideum* cell extracts a bacteriolytic activity effective against *Klebsiella pneumoniae* bacteria. In this study, we characterized bacteriolytic activities found in *D. discoideum* cell extracts against five different bacteria (*K. pneumoniae*, *Escherichia coli*, *Pseudomonas aeruginosa*, *Staphylococcus aureus*, and *Bacillus subtilis*). We first analyzed the bacteriolytic activity against these five bacteria in parallel over a range of pH values. We then measured the remaining bacteriolytic activity in *D. discoideum kil1* and *modA* knockout mutants. We also performed partial fractionation of *D. discoideum* extracts and assessed activity against different bacteria. Together our results indicate that optimal bacteriolytic activity against different bacteria results from the action of different effectors. Proteomic analysis allowed us to propose a list of potential bacteriolytic effectors.

**IMPORTANCE:**

Many antibacterial effectors have been characterized over the past decades, and their biological importance, mode of action, and specificity are often still under study. Here we characterized *in vitro* bacteriolytic activity in *D. discoideum* extracts against five species of Gram-negative and Gram-positive bacteria. Our results reveal that optimal lysis of different bacteria mobilizes different effectors. Proteomic analysis generated a list of potential bacteriolytic effectors. This work opens the way for future analysis of the role of individual effectors in living *D. discoideum* cells.

## INTRODUCTION

Phagocytic cells ingest bacteria, then kill and destroy them in phagocytic compartments. Intracellular killing and destruction of bacteria in phagosomes are a complex, multistep process. This process has been extensively studied in mammalian professional phagocytic cells (neutrophils and macrophages), which defend the body against bacterial infections. A large collection of molecular mechanisms has been proposed to play a role in intracellular killing and can be grouped in four general categories (1): toxic reactive oxygen or nitrogen species, specific ions (including protons), peptides capable of permeabilizing bacterial membranes, and enzymes digesting the bacterial constituents. Pathogenic bacteria have developed a vast array of evasion strategies to avoid capture and destruction by phagocytic cells both in the human body and in the environment (2).

*Dictyostelium discoideum* amoebae are found in forest soil, where they feed upon other soil microorganisms, particularly bacteria (2). *D. discoideum* cells are easy to handle, and their haploid genome is relatively easy to manipulate. *D. discoideum* has been used as a model to study many unicellular traits (e.g., motility, chemotaxis, or phagocytosis) as well as its starvation-induced multicellular development (3). More specifically, *D. discoideum* is a model phagocytic cell to study how phagocytic cells ingest, kill, and destroy bacteria in phagosomes. We analyzed previously the phenotypes of a collection of *D. discoideum* mutants and showed that different *D. discoideum* gene products are required to destroy different bacterial species in living cells (4). Our results also demonstrated that bacterial death of a non-pathogenic *Klebsiella pneumoniae* bacteria (KpGe strain) (5) in phagosomes precedes the permeabilization of its membrane(s) and its gradual dismantling by digestive enzymes (6).

Both in mammalian phagocytes and in *D. discoideum*, the list of known phagosomal bactericidal mechanisms is presumably incomplete, as well as their relative importance at different stages of the bacteriolytic process. It is also unclear whether different mechanisms are at play to ensure killing of different bacteria.

Antibacterial mechanisms can be studied following at least two distinct approaches. First, cellular gene products potentially implicated in intracellular killing can be genetically altered to evaluate their importance *in vivo*. Second, proteins with antibacterial activity can be identified *in vitro* in cellular extracts, purified, and characterized.

Previously, we showed that *D. discoideum* cell extracts exhibit a bacteriolytic activity against *K. pneumoniae* bacteria (7). A partially purified *D. discoideum* bacteriolytic fraction contained at least 16 proteins. In the current study, we analyzed the bacteriolytic activity of *D. discoideum* extracts against several Gram-negative and Gram-positive bacteria. Besides providing an extensive analysis of potential antibacterial proteins, our results indicate that optimal lysis of each of the five bacteria mobilizes different cellular proteins.

## RESULTS

### *D. discoideum* extracts exhibit bacteriolytic activity against five different bacteria

**Fig 1:**
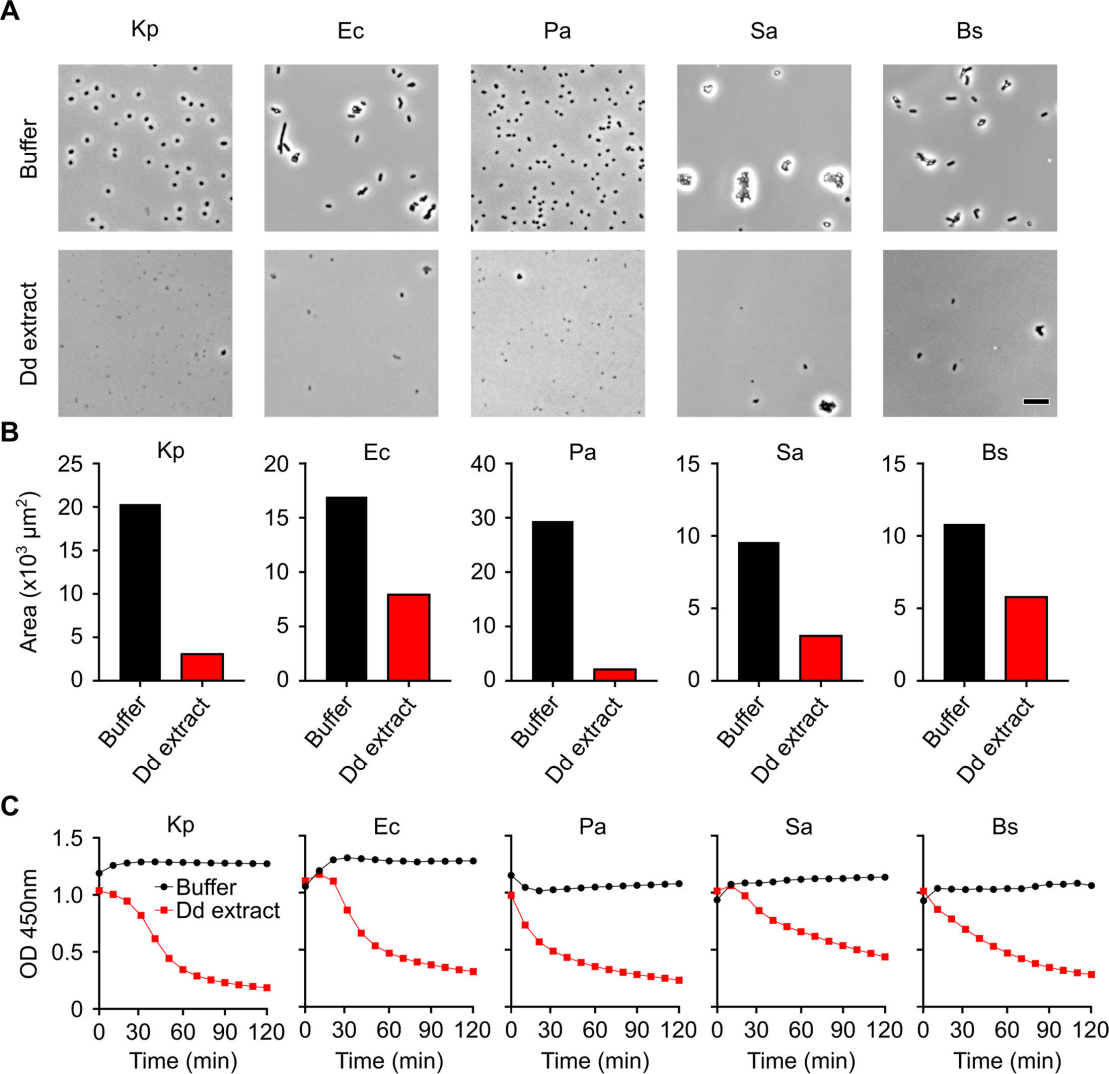
Assessing the bacteriolytic activity of *D. discoideum* extracts against different species of bacteria. (**A**) Different bacteria were incubated for 2 h in the presence (Dd extract) or absence (buffer) of unfractionated *D. discoideum* extracts. Bacterial lysis was assessed visually using phase contrast microscopy. (**B**) The area occupied by bacteria in pictures was quantified to evaluate the degree of bacterial lysis. (**C**) Bacterial lysis was followed by measuring the optical absorbance (OD) at 450 nm. Extracts from *D. discoideum* lysed all bacteria tested with similar efficiencies, and the lysis was most easily followed by measuring optical density. Bs, *B. subtilis*; Dd, *D. discoideum*; Ec, *E. coli*; Kp, *K. pneumoniae*; OD, optical density; Pa, *P. aeruginosa*; Sa, *S. aureus*.

We first assessed in parallel the bacteriolytic effect of *D. discoideum* extracts against three Gram-negative bacteria (*K. pneumoniae*, *Escherichia coli*, and *P. aeruginosa*), as well as two Gram-positive bacteria (*Staphylococcus aureus* and *Bacillus subtilis*). In order to compare the ability of *D. discoideum* cell extracts to lyse different types of bacteria, we mixed *D. discoideum* cell extracts with comparable numbers of bacteria of each bacterial species and visualized the bacteria after 2 h. Some bacteria (*K. pneumoniae* and *P. aeruginosa*) are easily visualized and counted, and they were both lysed with comparable efficiencies (Fig. 1A). For other bacteria (*E. coli*, *B. subtilis*, and to a much larger extent *S. aureus*), the presence of large bacterial aggregates prevented a precise counting of bacteria (Fig. 1A). However, in all cases, it was apparent that exposure to *D. discoideum* extracts resulted in a significant cell lysis. The amount of lysis was quantified by measuring in each picture the surface occupied by unlysed bacteria (Fig. 1B; Table S1). This quantification confirmed that *D. discoideum* cell extracts caused massive lysis of all five bacteria used in this study. Bacteria that aggregated were apparently less efficiently lysed than non-aggregating bacteria. This could reflect the fact that bacteria included in aggregates were less accessible to bacteriolytic effectors. Alternatively, the quantification method may underestimate the efficiency of lysis when applied to bacterial aggregates. Overall, it appears that different bacteria were lysed with comparable efficiencies by *D. discoideum* cell extracts. From a practical point of view, assessing the optical density of the bacterial suspension provided a measure of bacterial lysis consistent with microscopic analysis (Fig. 1C). Indeed, the optical density of the bacterial suspension decreased gradually as the bacteria were lysed. This method was used in the rest of this study to determine the bacteriolytic activity of different cellular extracts. Note that this assay measures lysis of bacteria, i.e., the permeabilization of the bacterial envelope and its dismantling by bacteriolytic proteins, not the loss of bacterial viability. In the experimental conditions described here, bacterial viability is largely compromised at pH values below 3 (for *E. coli* bacteria) or 4 (for other bacteria tested) (Fig. S1).

**Fig 2:**
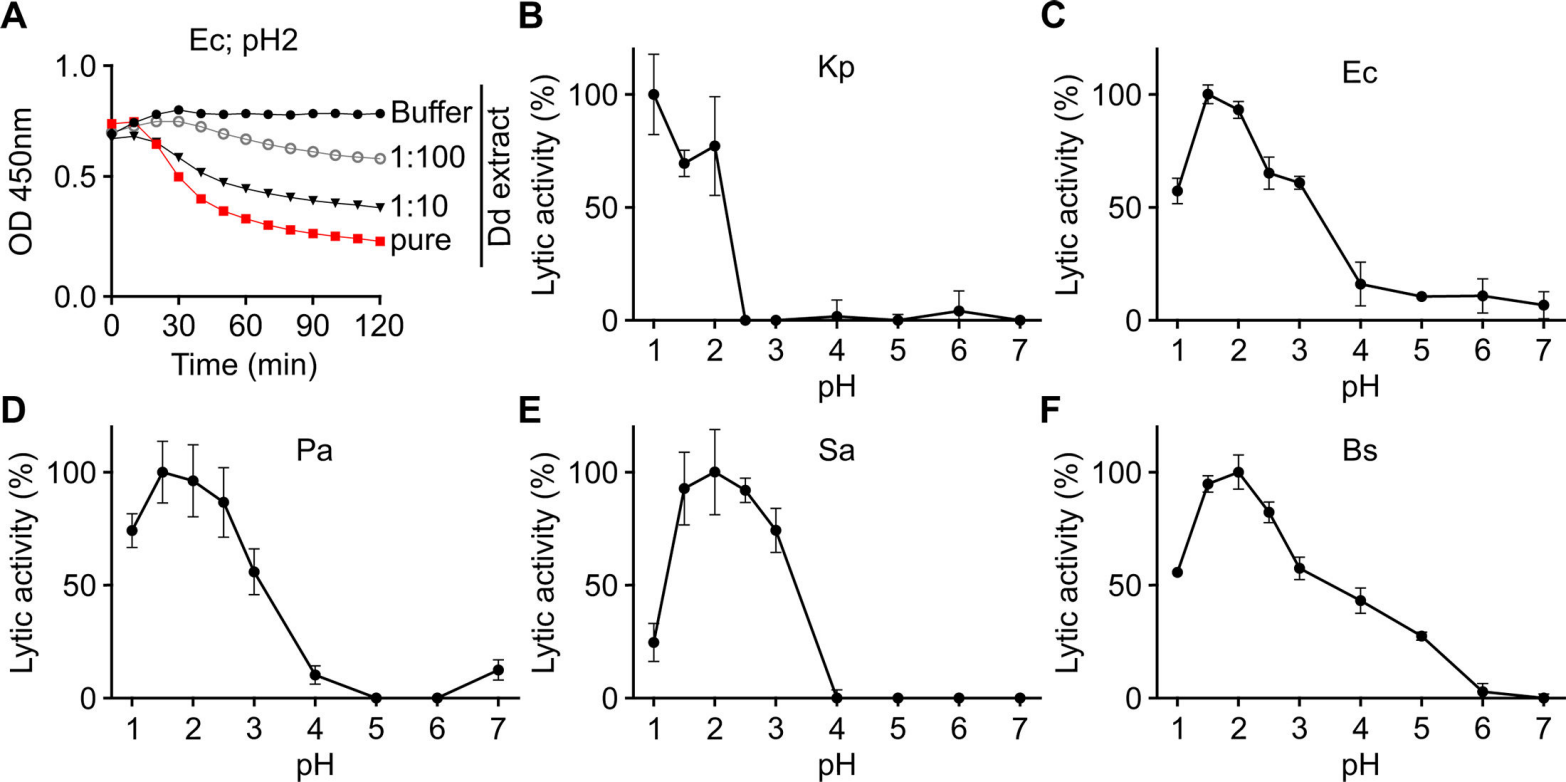
Antibacterial activity of *D. discoideum* extracts at different pH values. (**A**) In this example, wild-type *D. discoideum* cell extract was serially diluted (from 1/10 to 1/100) in lysis buffer and then mixed with *E. coli* (Ec) bacteria at pH 2. The bacteriolytic activity was monitored over time by spectrophotometry at 450 nm (OD_450_). (**B–F**) Antibacterial activity was determined over a range of pH against *K. pneumoniae* (Kp) (**B**), *E. coli* (Ec) (**C**), *P. aeruginosa* (Pa) (**D**),*S. aureus* (Sa) (**E**), and *B. subtilis* (Bs) (**F**). Mean ± SEM; *n* = 3 independent experiments for Kp, Ec, Sa, and Bs; *n* = 5 independent experiments for Pa. The method used to calculate the bacteriolytic activity is detailed in Fig. S6.

To obtain more quantitative estimates of bacteriolytic activity, bacterial lysis was quantified by testing the activity of several dilutions of *D. discoideum* extracts. In the example shown, the effect of pure or diluted *D. discoideum* extracts was tested on *E. coli* at pH 2 (Fig. 2A). As expected, bacterial lysis was less efficient as the cell extract was diluted, and no lysis was observed in buffer alone. We first used this assay to assess bacteriolytic activities over a range of pH. As previously described (7), *D. discoideum* extracts lysed efficiently *K. pneumoniae* at pH 1–2 (Fig. 2B). This very acidic pH reproduces the conditions found in the very acidic *D. discoideum* lysosomes and phago-lysosomes (8, 9). The bacteriolytic activity against *E. coli* (Fig. 2C), *P. aeruginosa* (Fig. 2D), *S. aureus* (Fig. 2E), and *B. subtilis* (Fig. 2F) was also optimal at very acidic pH (1.5–2.0). However, the activity profiles differed when different bacteria were lysed: while no lytic activity was seen against *K. pneumoniae* at pH 2.5 or higher, for the other four bacteria, a significant level of activity was still observed at pH 2.5 and 3.0. Only *B. subtilis* was lysed by *D. discoideum* extracts at a pH value of 4 or higher.

### Bacteriolytic activity in extracts from *D. discoideum* mutant cells

**Fig 3:**
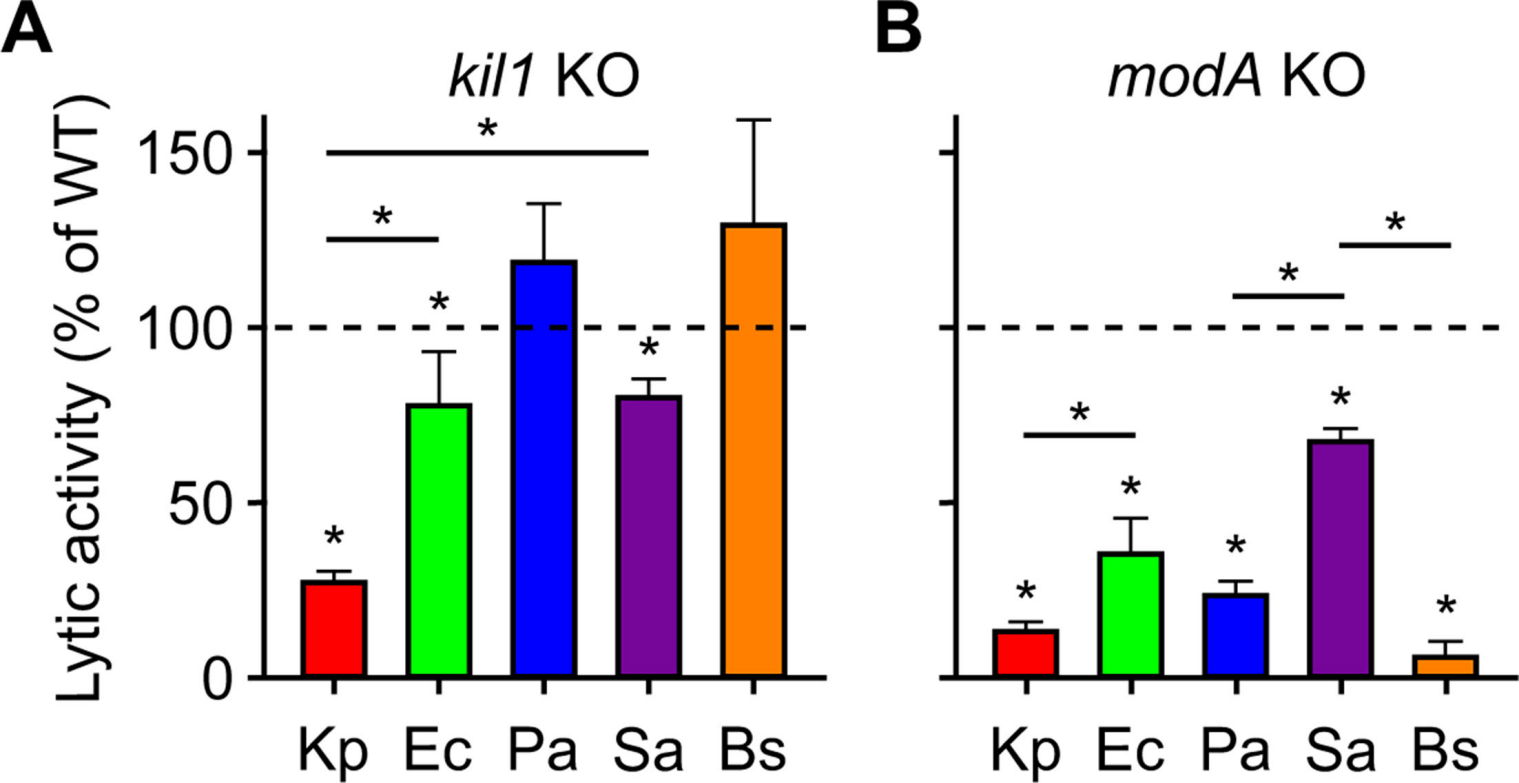
Lytic activity of *D. discoideum* extracts from *kil1* and *modA* KO cells. The bacteriolytic activity of *D. discoideum* extracts was tested as described in the legend of Fig. 2 at pH 2 against different bacteria. The activity of extracts from mutant (*kil1* KO and *modA* KO) cells was calculated as a percentage of the activity of extracts from WT cells, tested in parallel (represented by the dotted line at 100% activity). (**A**) Extracts from *kil1* KO cells exhibited a very reduced activity against *K. pneumoniae* (Kp) bacteria and a small but significant reduction in activity against *E. coli* (Ec) and *S. aureus* (Sa). Antibacterial activity against *P. aeruginosa* (Pa) and *B. subtilis* (Bs) was not significantly different in extracts from *kil1* KO cells and WT cells. (**B**) Extracts from *modA* KO cells lysed all bacteria less efficiently than extracts from WT cells. The effect was most pronounced against *K. pneumoniae*, *P. aeruginosa*, and *B. subtilis* bacteria, and less against *E. coli* and *S. aureus* bacteria. Mean ± SEM. *P < 0.05. Mann-Whitney test; *n* = 5 independent experiments for Kp and Sa, 9 for Pa and Bs, 10 for Ec exposed to *kil1* KO cell extracts; *n* = 5 independent experiments for all five bacteria exposed to *modA* KO cell extracts.

We next analyzed the bacteriolytic activity present in extracts from *kil1* knockout (KO) cells. Kil1 is the main sulfotransferase in *D. discoideum* cells, and *kil1* KO cells show a decreased ability to kill and destroy *K. pneumoniae* bacteria (6, 10) and, to a lesser extent, *E. coli* bacteria (4). As previously reported (7), extracts from *kil1* KO cells exhibited a strongly reduced ability to lyse *K. pneumoniae* bacteria (Fig. 3A). Lysis of *E. coli* and *S. aureus* bacteria by *kil1* KO extracts were also less efficient than with wild-type (WT) extracts, but the lysis was significantly weaker (Fig. 3A). Extracts from *kil1* KO cells lysed *P. aeruginosa* and *B. subtilis* as efficiently as extracts from WT cells (Fig. 3A).

We then tested extracts from a different *D. discoideum* mutant. ModA is a glycosidase present in the endoplasmic reticulum which plays a key role in the maturation and transport of many lysosomal enzymes. In *modA* KO cells, the activity of many lysosomal enzymes is significantly reduced (11). We generated *modA* KO cells as detailed in Materials and Methods and in Fig. S2. Extracts from *modA* KO cells lysed all five bacteria species much less efficiently than extracts from WT cells (Fig. 3B). The defect was very strong for lysis of *K. pneumoniae*, *P. aeruginosa*, and *B. subtilis*, and less prominent for the lysis of *E. coli* and *S. aureus*.

### Charge-based fractionation of *D. discoideum* bacteriolytic factors

**Fig 4:**
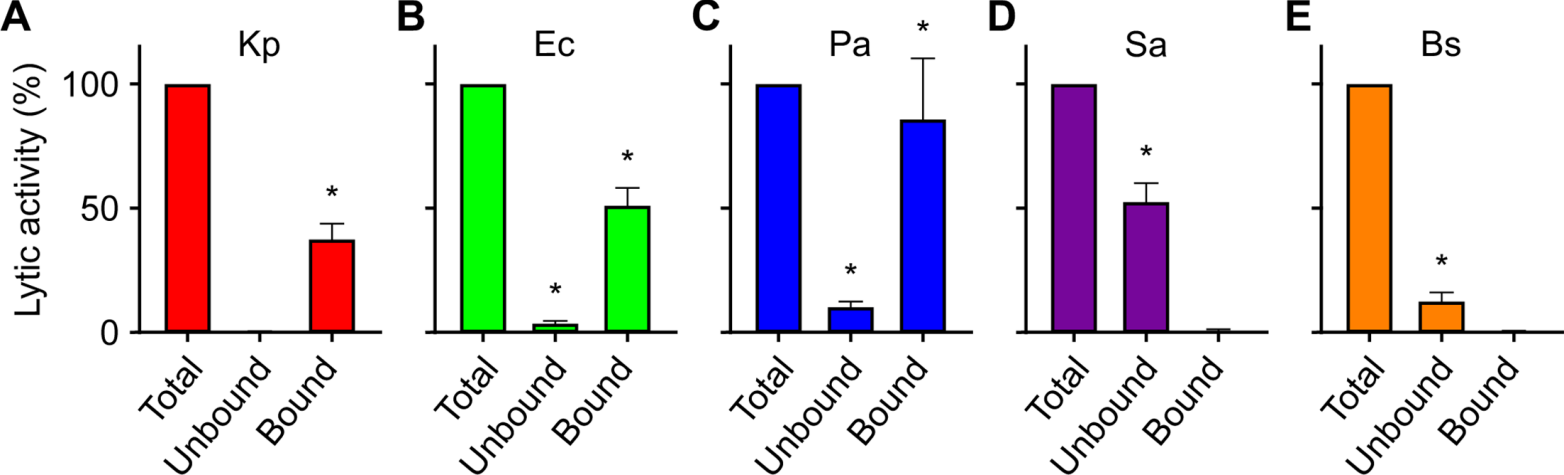
Initial fractionation of *D. discoideum* extracts. Extracts from *D. discoideum* (total) were incubated with an anion-exchange resin at pH 3.0. The supernatant (unbound) was collected. The resin was washed, then the bound proteins (bound) were eluted in 1M NaCl. The bacteriolytic activity of the different fractions was tested against *K. pneumoniae* (Kp) (**A**), *E. coli* (Ec) (**B**), *P. aeruginosa* (Pa) (**C**), S*. aureus* (Sa) (**D**), and *B. subtilis* (Bs) (**E**). Mean ± SEM. *Significantly positive value, *P* < 0.05. One-tailed Wilcoxon signed-rank test; *n* = 6 independent experiments for Kp, Ec, and Pa; *n* = 5 for Sa and Bs. The antibacterial activity was observed mostly in the bound fraction for Gram-negative bacteria and in the unbound fraction for Gram-positive bacteria.

As previously described, a large number of putative lysosomal enzymes can be purified based on their ability to bind to a positively charged resin at a very acidic pH (3.0) (7), presumably reflecting the fact that many lysosomal enzymes are decorated with sulfated or phosphorylated sugar moieties, which would remain negatively charged even at a very acidic pH (12). We used this property to separate *D. discoideum* cell extracts into two main fractions: the fraction bound to anion-exchange beads and the unbound fraction. For the three Gram-negative bacteria tested (*K. pneumoniae*, *E. coli*, and *P. aeruginosa*), bacteriolytic activity was detected in the fraction of proteins binding the anion-exchange resin and was entirely depleted from the unbound fraction (Fig. 4A through C). On the contrary, bacteriolytic activity against Gram-positive bacteria (*S. aureus* and *B. subtilis*) was found in the fraction of proteins not bound to the resin and absent from the fraction of bound proteins (Fig. 4D and E).

### Fractionation of bacteriolytic activity against Gram-negative bacteria

**Fig 5:**
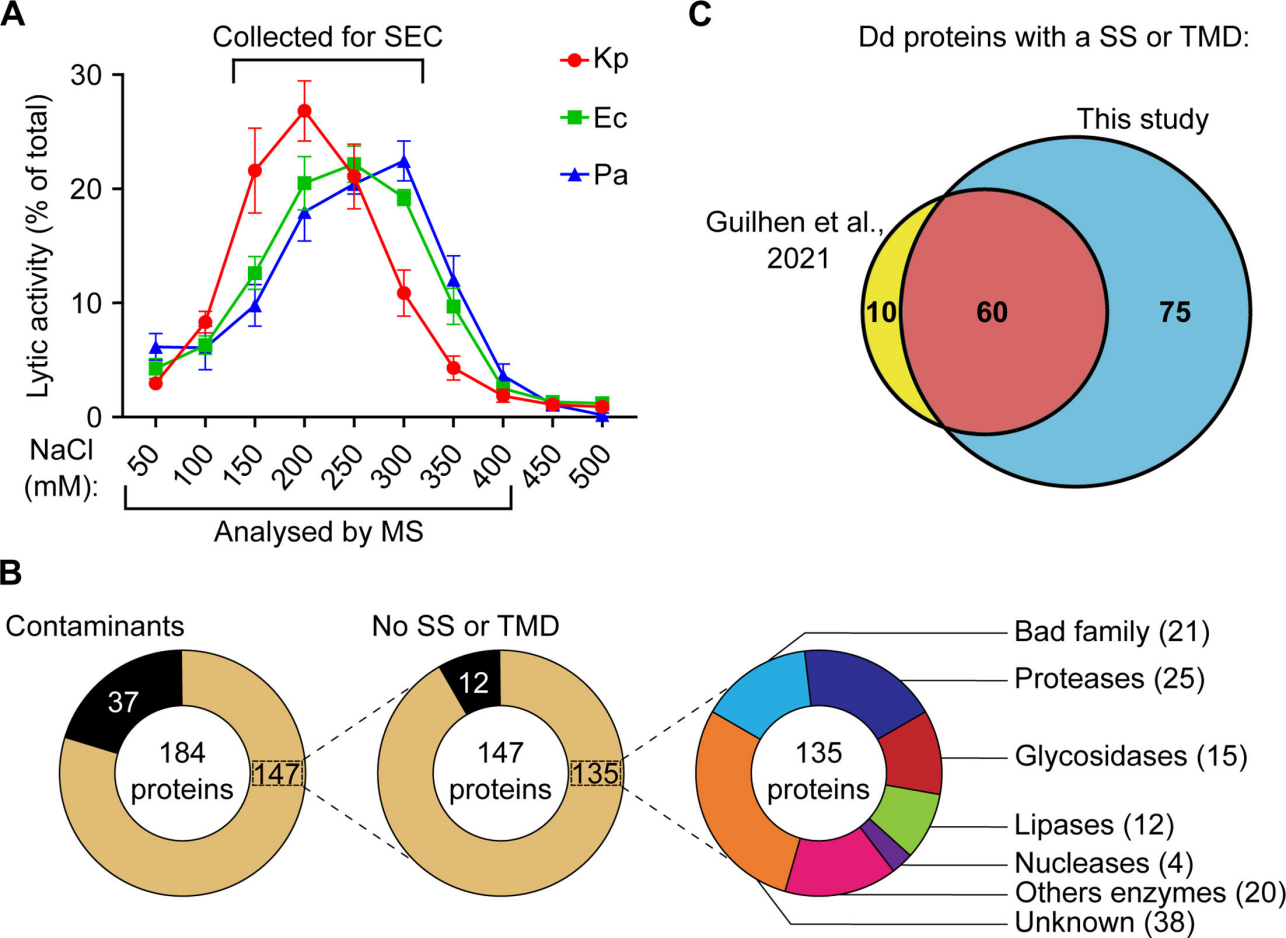
Separation of antibacterial proteins bound to an anion-exchange resin. Extracts from *D. discoideum* were incubated with an anion-exchange resin at pH 3. The resin was washed, then the bound proteins were eluted at increasing concentrations of NaCl. (**A**) The antibacterial activity of each fraction was tested against *K. pneumoniae* (Kp), *E. coli* (Ec), and *P. aeruginosa* (Pa). Activity against *K. pneumoniae* was most prominent in fractions eluted at lower salt concentrations (200-mM NaCl) than activity against *E. coli* (250 mM) and *P. aeruginosa* (300 mM). Mean ± SEM, *n* = 6 independent experiments. (**B**) The protein content of each fraction was analyzed by mass spectrometry and revealed the presence of a large number of presumptive lysosomal enzymes. The detailed list and abundance of different proteins with a signal sequence (SS) or a transmembrane domain (TMD) in each fraction are detailed in Table S3. (**C**) The list of proteins identified in the current study was compared with a similar earlier study (7). The list of proteins identified in the current study comprised the majority of proteins previously identified (60 proteins in common) plus a large set of previoulsy unidentified proteins (75 proteins). Dd, *D. discoideum*; MS, mass spectrometry.

We then purified further the proteins responsible for the observed bacteriolytic activity against gram-negative bacteria. For this, we eluted the proteins bound to the anion-exchange resin in the presence of increasing concentrations of NaCl and measured the bacteriolytic activity in each eluted fraction (Fig. 5A). The results of six independent experiments were pooled, allowing subtle differences to be reliably detected. Remarkably, the bacteriolytic activities against *K. pneumoniae*, *E. coli*, and *P. aeruginosa* were differently distributed in fractions eluted at different salt concentrations (Fig. 5A): the maximal lytic activity was found in the fraction eluted by 200-mM NaCl for *K. pneumoniae*, 250-mM NaCl for *E. coli*, and 300-mM NaCl for *P. aeruginosa*.

We used mass spectrometry to analyze the eight fractions exhibiting bacteriolytic activity (50- to 400-mM NaCl). As previously described (7), only a small set of *D. discoideum* proteins attached to an anion-exchange resin at acidic pH (Fig. 5B). A total of 147 *D. discoideum* proteins were detected in fractions eluted from the resin. Of these 147 proteins, 135 (92%) exhibited a putative signal sequence (SS) and/or transmembrane domain (TMD). Of these, a large number have a known or suspected lytic activity, and 38 (30%) have no known activity (Fig. 5B). A more detailed analysis of this set of proteins is presented below. This set of proteins includes the vast majority of the proteins detected in a similar previous study (7) (60 proteins), as well as a large number of previously undetected proteins (75 proteins) (Fig. 5C). This is presumably due to the fact that larger amounts of proteins were purified in the current study. A list of the 20 most abundant proteins bound to an anion-exchange resin is presented in Fig. S3.

**Fig 6:**
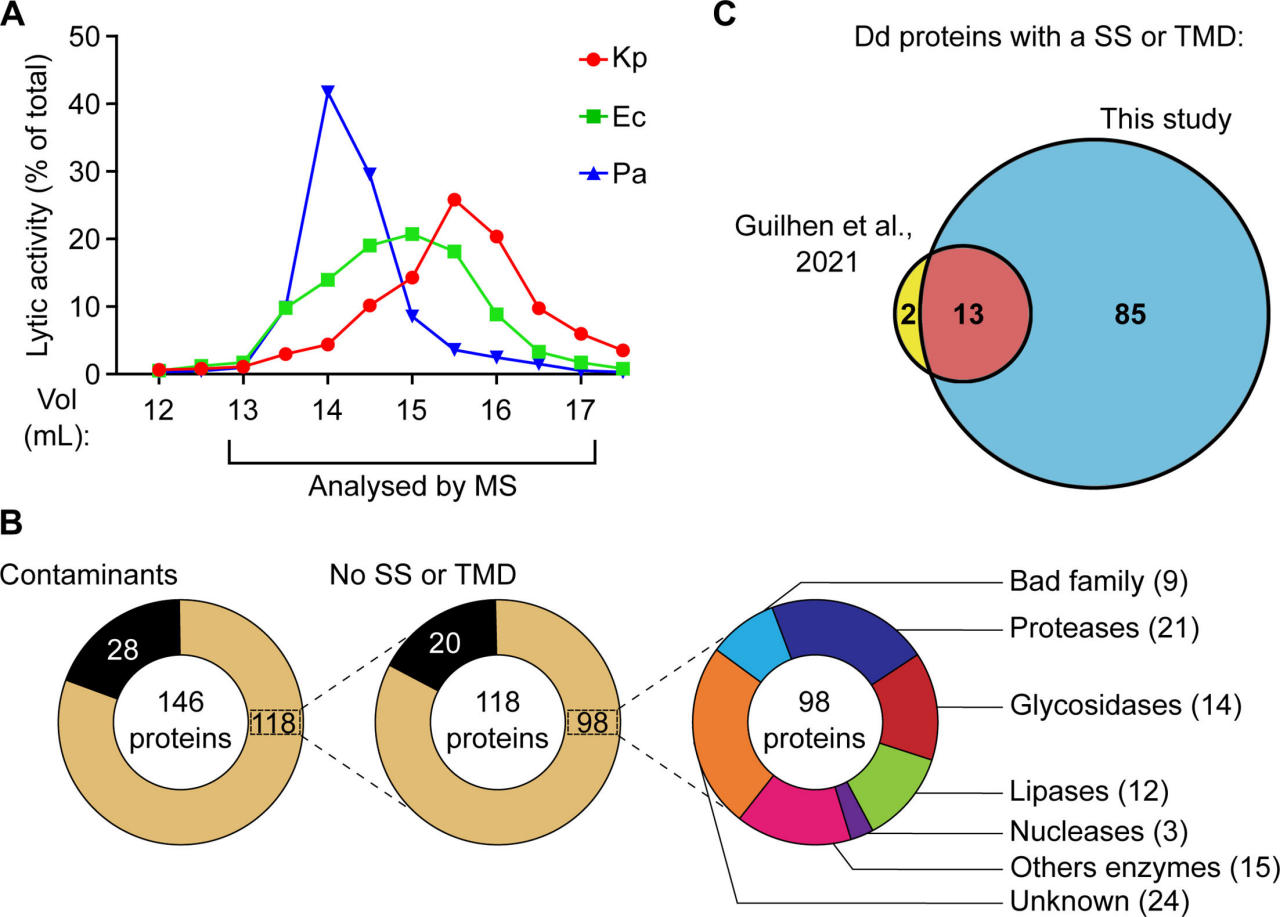
Size exclusion chromatography of antibacterial extracts. Fractions indicated in Fig. 5A were collected, mixed, and further separated on a size exclusion column. (**A**) A representative experiment shows that antibacterial activity against *K. pneumoniae* (Kp), *E. coli* (Ec), and *P. aeruginosa* (Pa) was most prominent in different fractions. Another example is shown in Fig. S4. (**B**) The protein content of each fraction was analyzed by mass spectrometry and revealed the presence of a large number of presumptive lysosomal enzymes. The detailed list and abundance of different proteins with a signal sequence (SS) or a transmembrane domain (TMD) in each fraction are detailed in Table S5. (**C**) The list of proteins identified in the current study comprised the majority of proteins previously identified (13 proteins in common) (7) plus a large set of previoulsy unidentified proteins (85 proteins). Dd, *D. discoideum*; MS, mass spectrometry.

**Fig 7:**
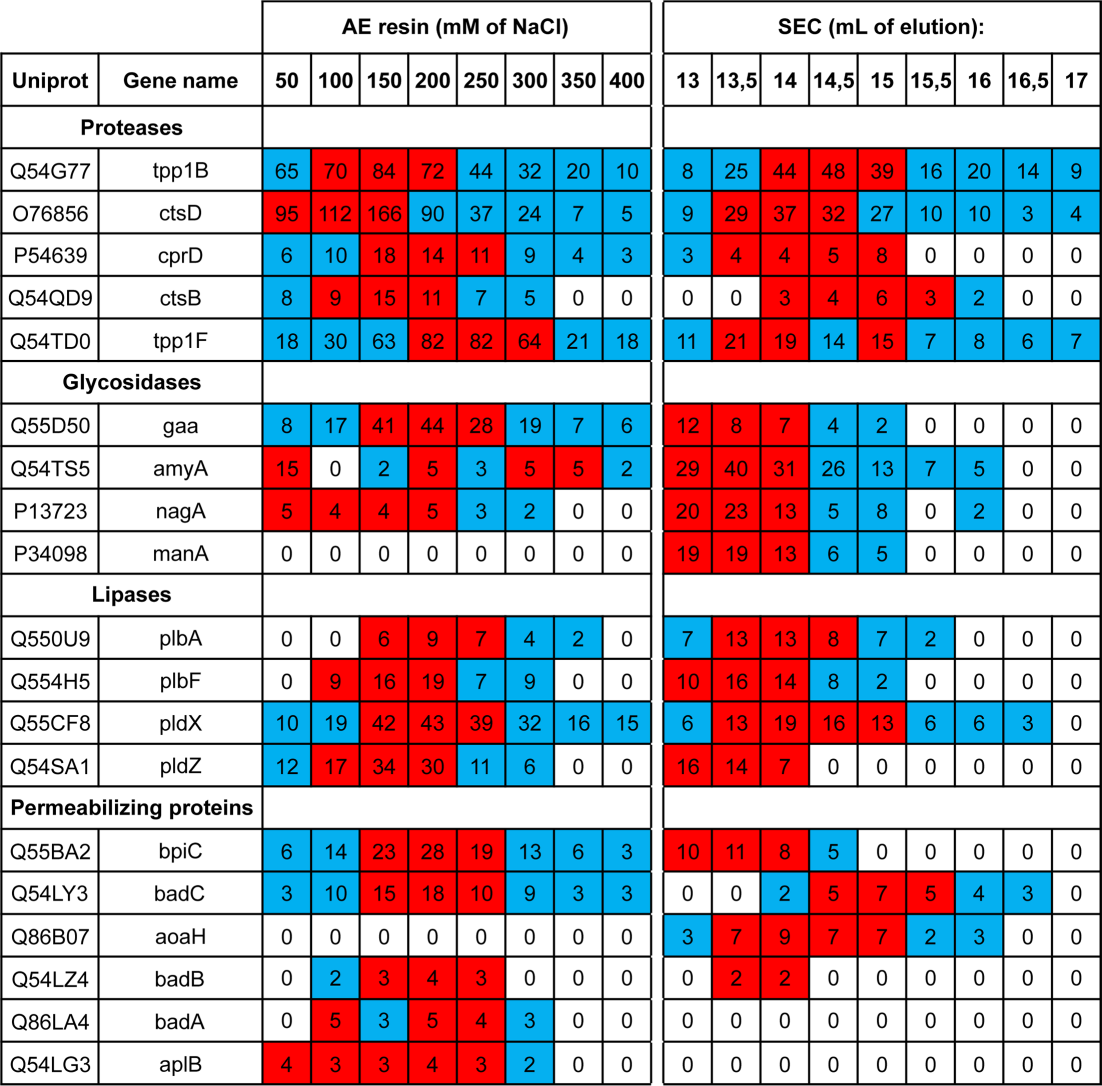
A list of candidate antibacterial effectors. A few putative proteases, glycosidases, lipases, and permeabilizing proteins were selected among the most abundant proteins detected in proteomics analysis. For each protein, the Uniprot number, as well as its gene name, and the number of peptides detected by mass spectrometry (spectrum count) in each fraction are indicated. For each protein, the three highest spectrum count values are highlighted in red, while the other positive spectrum counts are in blue. A complete set of all proteins with a signal sequence (SS) or a transmembrane domain (TMD) detected in this study is shown in Table S2. AE, anion exchange; SEC, size exclusion chromatography.

We further purified *D. discoideum* proteins by separating them based on their molecular weight on a gel filtration column. For this, we grouped the four fractions eluted at 150- to 300-mM NaCl and applied them on a Superdex 200 column. The lytic activity against *K. pneumoniae*, *E. coli*, and *P. aeruginosa* was then measured in each fraction (Fig. 6A). We observed again that the lytic activities against the three Gram-negative bacteria did not co-purify: the lytic activity against *P. aeruginosa* was more abundant in early fractions containing larger proteins (14-mL elution volume, estimated size 70 kDa), while for *E. coli*, it peaked in later fractions (15-mL elution volume, ≈40 kDa) and for *K. pneumoniae*, it was found in even later fractions (15.5–16.0 mL, ≈30 kDa) (Fig. 6A). Experimental variability prevented reliable averaging of multiple experiments, but other experiments showed very similar results (another example is shown in Fig. S4). Proteomic analysis of nine fractions (from 13 to 17 mL) detected the presence of a total of 98 *D. discoideum* proteins with an SS or a TMD. At least 66 of these 98 proteins (67%) exhibited a known or suspected lytic activity (Fig. 6B). This set of proteins is much larger than in a previous study (7) (Fig. 6C). A list of the 20 most abundant proteins purified after size exclusion chromatography is presented in Fig. S5. A selected set of the most abundant proteins identified in this study is shown, including proteases, glycosidases, lipases, and membrane-permeabilizing proteins (Fig. 7). Given the fact that bacteriolytic activity was observed in all fractions analyzed, some of these proteins, separately or in combination, must be able to lyse Gram-negative bacteria. The complete list of all *D. discoideum* proteins with SS or a TMD detected in this study is shown in Table S2.

## DISCUSSION

**Fig 8:**
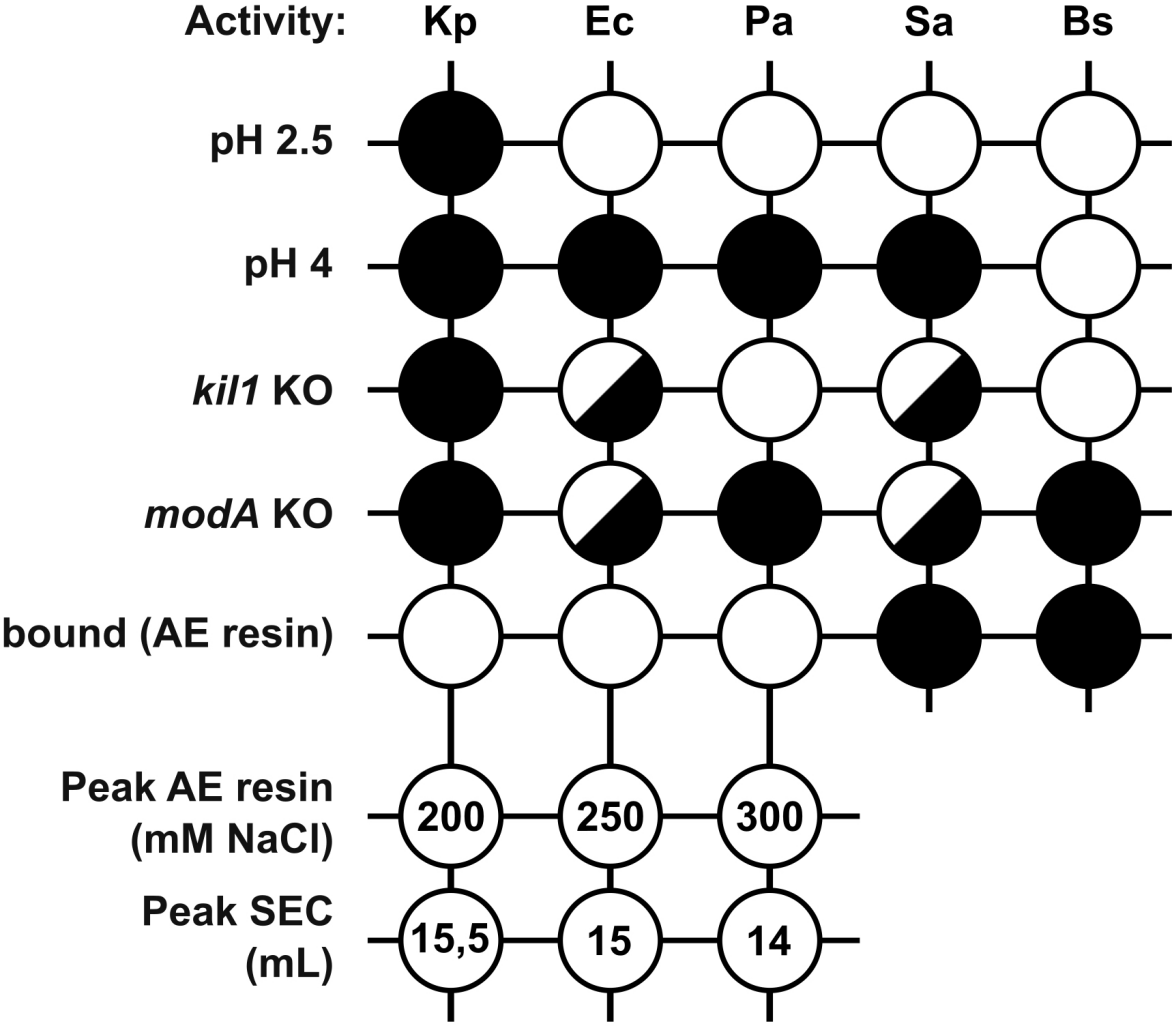
Summary of experimental results. *D. discoideum* cell extracts lyse all five bacteria tested in optimal conditions (pH 1–2). Differences appeared in other conditions (pH 2.5 and 4.0) when extracts from mutant cells (*kil1* KO and *modA* KO) were used, when only proteins binding an anion-exchange (13) resin were tested, or when these proteins were further purified by gradual elution from anionic exchange (13) resin and by size exclusion chromatography (SEC). Overall, these results reveal that lysis of each bacteria tested is affected differentially in different conditions, indicating that different effectors are mobilized for optimal lysis of each bacteria. White circles denote efficient bacterial lysis; split circles indicate reduced bacterial lysis; black circles denote virtually abolished bacterial lysis. For elution from AE resin and SEC, the fraction containing the highest level of bacteriolytic activity is indicated.

In this study, we show that *D. discoideum* extracts exhibit lytic activity against five different bacteria, three Gram negative (*K. pneumoniae*, *E. coli*, and *P. aeruginosa*) and two Gram positive (*S. aureus* and *B. subtilis*). Together, our results suggest that different molecular mechanisms lead to the lysis of these five bacteria, as summarized graphically in Fig. 8. First, the lytic activity against each bacteria presented a varying degree of susceptibility to pH. The lysis of all five bacteria was optimal at very acidic pH levels. Lysis of *K. pneumoniae* was only observed at a pH level of 2 or lower. In contrast, other bacteria were still lysed at higher pH levels: *E. coli*, *P. aeruginosa*, and *S. aureus* were lysed at pH 2.5–3, and *B. subtilis* at pH four or higher. Second, genetic inactivation of *kil1* strongly reduced the lytic activity against *K. pneumoniae*, decreased moderately the lytic activity against *E. coli* and *S. aureus*, and did not significantly affect the lytic activity against *P. aeruginosa* and *B. subtilis*. Third, genetic inactivation of *modA* produced an entirely different pattern: the remaining activity against *K. pneumoniae*, *B. subtilis* and *P. aeruginosa* bacteria was very low, while it was higher against *E. coli* and *S. aureus*. This indicates varying degrees of dependency to either *kil1* or *modA*-related mechanisms in between all five bacteria. Loss of Kil1 is expected to reduce the activity of a subset of sulfated lysosomal proteins, whereas loss of ModA perturbs the maturation and transport of a large spectrum of glycosylated lysosomal proteins, presumably accounting for their differential effects. Fourth, the lytic activity against Gram-negative bacteria (*K. pneumoniae*, *E. coli*, and *P. aeruginosa*) was readily detected in a fraction of proteins bound to an anion-exchange resin, while the activity against Gram-positive bacteria (*S. aureus* and *B. subtilis*) was not. Fifth, proteins bound to an anion-exchange resin were eluted stepwise, and the optimal bacteriolytic activity against *K. pneumoniae*, *E. coli*, and *P. aeruginosa* was found in different fractions. Sixth, proteins were separated further on a gel filtration column, and again optimal activity against *K. pneumoniae*, *E. coli*, and *P. aeruginosa* was found in different fractions. Together these results clearly indicate that different proteins are required for optimal lysis of the five bacteria tested here.

Importantly, in the current study, we did not reach the degree of purity that would allow assessment of the bactericidal activity of single proteins, and our attempts to purify further bacteriolytic proteins actually led to a loss of the activity. It is likely that in our experiments, bactericidal activity against different bacteria was achieved by the combined activity of several proteins.

Our results obtained *in vitro* are compatible with previous observations in living cells. Indeed, in living cells, genetic inactivation of genes encoding putative lytic effectors like *bpiC* reduced and delayed intracellular destruction of some bacteria (*E. coli* and to a lesser extent *K. pneumoniae* and *P. aeruginosa*) but not others (*S. aureus* and *B. subtilis*) (4). Moreover, like in all mutants studied so far, the effect of the *bpiC* genetic inactivation was partial, and all ingested bacteria were eventually destroyed, albeit more slowly. These observations suggest that a specific mixture of proteins ensures the destruction of each bacteria in phagosomes, that none of them is essential and that their effect is at least in part cumulative. Ultimately, a combination of biochemical and genetic approaches will probably be ideal to fully define the mechanisms at play during bacterial destruction in phagosomes. One of the advantages of biochemical analysis is to identify new proteins potentially implicated in the bacterial destruction in phagosomes. The current study notably provides a short list of proteins potentially implicated in this process, which represent promising candidates for further analysis.

## MATERIALS AND METHODS

### *D. discoideum* culture and strains

*D. discoideum* DH1-10 parental strain (14), referred to for simplicity as WT, as well as *kil1* (10) and *modA* KO (this study) strains were cultured in HL5 medium (15) at 21°C in suspension and diluted twice a week. Cells were collected at a cellular density of 2 to 5 × 10^6^ cells/mL. The *modA* KO cells were created using CRISPR/Cas9 technology as described previously (16). Briefly, two sgRNA sequences were chosen using the website http://www.rgenome.net/cas-designer/. Each sgRNA was cloned into the pTM1285 plasmid using the Golden Gate assembly and the BpiI enzyme before transformation in TOP10 *E. coli*. Plasmids were purified using a maxiprep kit (Macherey-Nagel, #740574.25) and sequenced. *D. discoideum* cells (16 × 10^6^ cells in 400 µL) were electroporated with the pTM1285/sgRNA plasmid (20 µg) using a Bio-Rad Electroporator (Gene Pulser Xcell System) (17). Transfected *D. discoideum* cells were resuspended in 35 mL of HL5 medium. The next day, 15 µg/mL of G418 was added to the medium to select plasmid-containing cells. Six days later, surviving *D. discoideum* cells were cloned by limiting dilution in the absence of G418 in 96-well plates. To analyze individual clones, the genomic region of interest was amplified by PCR and sequenced. Two clones presenting different mutations were kept for further analysis. The detailed description of the *modA* KO mutant generated and analyzed in this study is found in Fig. S2.

### Bacterial culture and strains

The bacterial strains used were *K. pneumoniae* KpGe (5), *E. coli* REL606 (18), *P. aeruginosa* PT531 (19), non-sporulating *B. subtilis* strain 36.1 (20), and *S. aureus* ATCC 29213. Bacteria were grown overnight in lysogeny broth (LB) agar plates at 37°C. Before each experiment, a single colony was picked from the plate, inoculated into 3 mL of liquid culture medium (LB for *K. pneumoniae* and *S. aureus*, standard medium [SM] for *P. aeruginosa* and *B. subtilis*, and SM supplemented with 10 g/L of glucose for *E. coli*) (15) and grown for 16 h under agitation at 37°C.

### Bacterial lysis assessed by measuring optical density

To measure bacterial lysis, 1 × 10^8^ *D. discoideum* cells were washed twice in phosphate buffer (PB: 2 mM of Na_2_HPO_4_ and 14.7 mM of KH_2_PO_4_, pH 6.3) and lysed in 1 mL of lysis buffer (50-mM NaPO_4_ buffer, pH5, 0.5% Triton X-100) for 20 min at 4°C with occasional vortexing (20–30 s, every 5 min). The cell lysate was centrifuged (30,000 × *g*, 20 min at 4°C) to remove unlysed cell debris and nuclei, and the supernatant was collected. The cleared supernatant was buffered at the desired pH using HCl 1 M or NaOH 5 N, then diluted using lysis buffer at the desired pH (from 1 to 7). Bacteriolytic activity was assessed by mixing in a 96-well microtiter plate 100 µL of cell extract (or lysis buffer as a negative control) with 100 µL of bacterial culture washed once in 50-mM NaPO_4_ buffer at the corresponding pH and resuspended to a final optical density at 450 nm of 0.8–1.2. The decrease in turbidity at 21°C (optical density at 450 nm) was measured every 10 min for 2 h, with a spectrophotometer plate reader (Hidex Sense Plate Reader Software, version 1.3.0). To calculate the bacteriolytic activity of a sample, the OD_450_ values were normalized to the value of the sample at time 0 defined as 100%. The normalized OD_450_ values were then plotted as a function of time, and the area under the curve was calculated. The activity was then calculated by comparing with a standard curve obtained by diluting WT *D. discoideum* cell lysates. The calculation method used is detailed in Fig. S6.

### Bacterial viability at different pH

*K. pneumoniae*, *E. coli*, *P. aeruginosa*, *S. aureus*, and *B. subtilis* were grown overnight in liquid cultures of 3 mL as described above, then 500 µL of bacterial culture was pelleted by centrifugation (3 min, 5,000 g). The pellets were resuspended in NaPO_4_ buffer at a pH level ranging from 1 to 7. After 2 h, serial dilutions (15-µL droplets) were deposited on LB agar plates, and bacteria were grown overnight at 37°C (Fig. S1) .

### Bacterial lysis assessed by direct observation

To visualize bacterial lysis, bacteria were exposed to *D. discoideum* cell extracts as described above for 2 h, then 4 µL was placed on a glass slide and covered with a glass coverslip. The bacteria were imaged using a Zeiss Axio Observer Z1 with Definite Focus 2 microscope and an EC Plan-Neofluar ×20/0.50, Ph2 WD 2.0-mm objective. To quantify the degree of lysis, raw TIFF phase contrast images were processed using MATLAB R2023a (The MathWorks). Initially, a flat-field correction was applied, followed by a non-local mean filter on all images. Subsequently, these processed images were analyzed using QuPath (version 0.5.0) (21), where an artificial neural network was employed to classify pixels corresponding to bacteria within the field of view. This automated detection of bacteria was also corrected manually. For each sample, three separate images (each 624 × 501 µm) were analyzed to determine the number and size of bacterial particles. The particles were classified as “debris” (inferior to 0,9 µm^2^, not taken into account in calculations), “single bacteria” (between 0.9 and 2.0 µm^2^) or aggregates (superior to 2 µm^2^), as detailed in Table S1.

### Biochemical fractionation

*D. discoideum* extracts were fractionated essentially as described (7). Briefly, *D. discoideum*-cleared cell lysates described above were buffered at pH 3, then mixed with 200 µL of anion-exchange resin (Q Sepharose Fast Flow, Sigma #17–0510-10) pre-washed in lysis buffer at pH 3. After 1 h of incubation at 4°C on a rotating wheel, the anionic resin was pelleted (30,000 × *g* for 30 s at 4°C), and the supernatant was collected (unbound fraction). The resin was then washed five times with 1 mL of lysis buffer at pH 3. Negatively charged proteins attached to the resin were eluted at pH 3 in lysis buffer containing 1 M of NaCl (bound fraction). The bacteriolytic activity of total cell extracts, as well as unbound and bound fractions, was then assessed as described above.

To purify further the bacteriolytic activity, a more concentrated cell extract was used (5 × 10^8^ *D. discoideum* cells lysed in 1.75 mL of lysis buffer), and negatively charged proteins attached to the resin were eluted at increasing concentrations of NaCl, from 50 to 500 mM of NaCl with increments of 50 mM. Recovered fractions were tested for the presence of bacteriolytic activity. Fractions presenting the highest bacteriolytic activity (e.g., fractions 150, 200, 250, and 300 mM of NaCl) were mixed, buffered at pH 7, and loaded on a size-exclusion chromatographic (SEC) column (Superdex 200 10/300 Gl, Sigma #17–5175-01). The column was equilibrated with NaP buffer at pH 7 and eluted with the same buffer at a rate of 18 mL/h. Fractions of 0.5 mL were collected, and their bacteriolytic activity was tested.

### Protein identification by ESI-LC-MSMS

Proteins were identified as previously described (7). Briefly, protein samples were purified using one-dimensional gel electrophoresis, reduced, alkylated, and rehydrated with a solution containing trypsin, which digested proteins into peptides. Peptides were then injected into a column. Electrospray ionization-liquid chromatography-mass spectrometry was performed on a Q-Exactive HF Hybrid Quadrupole-Orbitrap Mass Spectrometer (Thermo Scientific) equipped with an Easy nLC 1000 liquid chromatography system (Thermo Scientific). Peptides were trapped on an Acclaim pepmap100, C18, 3 µm, 75 µm × 20 mm nano-trap column (Thermo Scientific) and separated on a 75 µm × 250 mm, C18, 2 µm, 100-Å Easy-Spray column (Thermo Scientific). Peptides were eluted and analyzed by mass spectrometry. The generated peak lists (MS Convert conversion tool from ProteoWizard) were searched against the *Dictyostelium discoideum* Reference Database (Uniprot, release 2020–05; 12,746 entries) combined with an in-house database of common contaminant using Mascot (version 2.5.1; Matrix Science, London, UK). The search results were validated using Scaffold (version 4.11.1, Proteome Software) and the Protein Prophet algorithm (13).

## Data Availability

The data sets used and/or analyzed during the current study are publicly available on an open access server (Yareta) under https://doi.org/10.26037/yareta:oro3nohevfcglcmhyd3hoiefkm.

## SUPPLEMENTAL MATERIAL

Figure S1 msphere.00471-24-s0001.eps Bacterial viability of different species of bacteria at acidic pH.

Figure S2 msphere.00471-24-s0002.eps Generation of *modA* KO cells using CRISPR/Cas9.

Figure S3 msphere.00471-24-s0003.eps Most abundant proteins bound to an anion exchange resin.

Figure S4 msphere.00471-24-s0004.eps Size exclusion chromatography of *D. discoideum* extracts.

Figure S5 msphere.00471-24-s0005.eps Most abundant proteins collected after separation on a size exclusion column.

Figure S6 msphere.00471-24-s0006.eps Determination of bacterial lysis activity.

Legends msphere.00471-24-s0007.docx Supplemental figure and table legends.

Table S1 msphere.00471-24-s0008.docx Bacterial count to assess the bacteriolytic activity of *D. discoideum* extracts.

Table S2 msphere.00471-24-s0009.docx Full list of proteins with a SS or TMD detected in this study.

ASM does not own the copyrights to Supplemental Material that may be linked to, or accessed through, an article. The authors have granted ASM a non-exclusive, world-wide license to publish the Supplemental Material files. Please contact the corresponding author directly for reuse.

## References

[1] Uribe-Querol E, Rosales C. 2017. Control of phagocytosis by microbial pathogens. Front Immunol 8:1368. doi:10.3389/fimmu.2017.01368 29114249 PMC5660709

[2] Cosson P, Soldati T. 2008. Eat, kill or die: when amoeba meets bacteria. Curr Opin Microbiol 11:271–276. doi:10.1016/j.mib.2008.05.005 18550419

[3] Bozzaro S. 2013. The model organism Dictyostelium discoideum. Methods Mol Biol 983:17–37. doi:10.1007/978-1-62703-302-2_2 23494300

[4] Jauslin T, Lamrabet O, Crespo-Yañez X, Marchetti A, Ayadi I, Ifrid E, Guilhen C, Leippe M, Cosson P. 2021. How phagocytic cells kill different bacteria: a quantitative analysis using Dictyostelium discoideum. mBio 12:e03169-20. doi:10.1128/mBio.03169-20 33593980 PMC8545105

[5] Lima WC, Pillonel T, Bertelli C, Ifrid E, Greub G, Cosson P. 2018. Genome sequencing and functional characterization of the non-pathogenic Klebsiella pneumoniae KpGe bacteria. Microbes Infect 20:293–301. doi:10.1016/j.micinf.2018.04.001 29753816

[6] Crespo-Yanez X, Oddy J, Lamrabet O, Jauslin T, Marchetti A, Cosson P. 2023. Sequential action of antibacterial effectors in Dictyostelium discoideum phagosomes. Mol Microbiol 119:74–85. doi:10.1111/mmi.15004 36416195 PMC10107278

[7] Guilhen C, Lima WC, Ifrid E, Crespo-Yañez X, Lamrabet O, Cosson P. 2020. A new family of bacteriolytic proteins in Dictyostelium discoideum. Front Cell Infect Microbiol 10:617310. doi:10.3389/fcimb.2020.617310 33614529 PMC7886984

[8] Marchetti A, Lelong E, Cosson P. 2009. A measure of endosomal pH by flow cytometry in Dictyostelium. BMC Res Notes 2:7. doi:10.1186/1756-0500-2-7 19138423 PMC2632630

[9] Bodinier R, Leiba J, Sabra A, Jauslin TN, Lamrabet O, Guilhen C, Marchetti A, Iwade Y, Kawata T, Lima WC, Cosson P. 2020. LrrkA, a kinase with leucine-rich repeats, links folate sensing with Kil2 activity and intracellular killing. Cell Microbiol 22:e13129. doi:10.1111/cmi.13129 31652367 PMC7003747

[10] Benghezal M, Fauvarque MO, Tournebize R, Froquet R, Marchetti A, Bergeret E, Lardy B, Klein G, Sansonetti P, Charette SJ, Cosson P. 2006. Specific host genes required for the killing of Klebsiella bacteria by phagocytes. Cell Microbiol 8:139–148. doi:10.1111/j.1462-5822.2005.00607.x 16367873

[11] Freeze HH, Lammertz M, Iranfar N, Fuller D, Panneerselvam K, Loomis WF. 1997. Consequences of disrupting the gene that encodes alpha-glucosidase II in the N-linked oligosaccharide biosynthesis pathway of Dictyostelium discoideum. Dev Genet 21:177–186. doi:10.1002/(SICI)1520-6408(1997)21:3<177::AID-DVG1>3.0.CO;2-4 9397534

[12] Freeze HH. 1986. Modifications of lysosomal enzymes in Dictyostelium discoideum. Mol Cell Biochem 72:47–65. doi:10.1007/BF00230635 2434832 PMC7089276

[13] Nesvizhskii AI, Keller A, Kolker E, Aebersold R. 2003. A statistical model for identifying proteins by tandem mass spectrometry. Anal Chem 75:4646–4658. doi:10.1021/ac0341261 14632076

[14] Cornillon S, Pech E, Benghezal M, Ravanel K, Gaynor E, Letourneur F, Brückert F, Cosson P. 2000. Phg1p is a nine-transmembrane protein superfamily member involved in Dictyostelium adhesion and phagocytosis. J Biol Chem 275:34287–34292. doi:10.1074/jbc.M006725200 10944536

[15] Froquet R, Lelong E, Marchetti A, Cosson P. 2009. Dictyostelium discoideum: a model host to measure bacterial virulence. Nat Protoc 4:25–30. doi:10.1038/nprot.2008.212 19131953

[16] Sekine R, Kawata T, Muramoto T. 2018. CRISPR/Cas9 mediated targeting of multiple genes in Dictyostelium. Sci Rep 8:8471. doi:10.1038/s41598-018-26756-z 29855514 PMC5981456

[17] Alibaud L, Cosson P, Benghezal M. 2003. Dictyostelium discoideum transformation by oscillating electric field electroporation. Biotechniques 35:78–80, doi:10.2144/03351st03 12866409

[18] Adiba S, Nizak C, van Baalen M, Denamur E, Depaulis F. 2010. From grazing resistance to pathogenesis: the coincidental evolution of virulence factors. PLoS One 5:e11882. doi:10.1371/journal.pone.0011882 20711443 PMC2920306

[19] Köhler T, Curty LK, Barja F, van Delden C, Pechère JC. 2000. Swarming of Pseudomonas aeruginosa is dependent on cell-to-cell signaling and requires flagella and pili. J Bacteriol 182:5990–5996. doi:10.1128/JB.182.21.5990-5996.2000 11029417 PMC94731

[20] Ratner DI, Newell PC. 1978. Linkage analysis in Dictyostelium discoideum using multiply marked tester strains: establishment of linkage group VII and the reassessment of earlier linkage data. J Gen Microbiol 109:225–236. doi:10.1099/00221287-109-2-225 745003

[21] Bankhead P, Loughrey MB, Fernández JA, Dombrowski Y, McArt DG, Dunne PD, McQuaid S, Gray RT, Murray LJ, Coleman HG, James JA, Salto-Tellez M, Hamilton PW. 2017. QuPath: open source software for digital pathology image analysis. Sci Rep 7:16878. doi:10.1038/s41598-017-17204-5 29203879 PMC5715110

