# Supplementary material for: Antibacterial effectors in Dictyostelium discoideum: specific activity against different bacterial species
Source: mSphere. 2024 Oct 8;9(10):e00471-24. doi: 10.1128/msphere.00471-24 (PMC11520349; doi:10.1128/msphere.00471-24)
Supplement: Legends — Supplemental figure and table legends. [file msphere.00471-24-s0007.docx]

**ADDITIONAL FILES**

**Figure S1. Bacterial viability of different species of bacteria at acidic pH.** *K. pneumoniae* (Kp), *E. coli* (Ec), *P. aeruginosa* (Pa), *S. aureus* (Sa) and *B. subtilis* (Bs) were incubated in NaPO_4_ buffer at different pH (from 1 to 7) for 2h, then serially diluted, deposited on an LB plate and grown overnight.

**Figure S2. Generation of *modA* KO cells using CRISPR/Cas9.** (**A**) Schematic representation of the *modA* gene and protein in WT and in KO cells. The sequence targeted by the guide RNA is indicated in red and the arrow indicates the cutting site of the Cas9 nuclease. The sequences of two independent guide RNAs and mutant clones are represented. The truncated and aberrant proteins predicted to be produced in mutant clones are shown. (**B**) Oligonucleotides used for the PCR amplification of the targeted genomic region.

**Figure S3. Most abundant proteins bound to an anion exchange resin .** The 20 most abundant *D. discoideum* proteins with a signal sequence (SS) or a transmembrane domain (TMD) binding an anion exchange resin (Fig. 5) are listed. For each protein, the Uniprot number, as well as its gene name, and the number of peptides detected by mass spectrometry (spectrum count) in each fraction are indicated. For each protein, the 3 highest spectrum count values are highlighted in red while the other positive spectrum count are in blue. AE: anion exchange; SEC: size exclusion chromatography

**Figure S4. Size exclusion chromatography of *D. discoideum* extracts.** This figure presents an experiment identical to the experiment shown in Fig. 6A. Like the data presented in Fig. 6A, this experiment indicates that the peak of antibacterial activity against *K. pneumoniae* (Kp), *E. coli* (Ec) and *P. aeruginosa* (Pa) was observed in different fractions.

**Figure S5. Most abundant proteins collected after separation on a size exclusion column.** The 20 most abundant proteins after purification by size exclusion chromatography (Fig. 6) are listed. For each protein, the Uniprot number, as well as its gene name, and the number of peptides detected by mass spectrometry (spectrum count) in each fraction are indicated. For each protein, the 3 highest spectrum count values are highlighted in red while the other positive spectrum count are in blue. AE: anion exchange; SEC: size exclusion chromatography

**Figure S6. Determination of bacterial lysis activity.** (**A**) Raw optical density (OD_450nm_) values were measured every 10 min over 2 h for a sample of interest (here *kil1* KO cell lysate), a standard range of dilution of WT cell lysate and a control condition (buffer). Raw OD_450nm_ values were normalized by setting the value of each sample at time 0 to 100% (**B**) and plotted into curves (**C**). Area under the curves (AUC) were calculated and normalized (“Norm AUC”) by dividing them by the AUC of the buffer condition. (**D**) Norm AUCs of the standard WT cell lysate samples were plotted to generate a calibration curve. (**E**) The equation fitting this standard curve was determined and used to calculate the percentage of activity of the sample of interest (here 78%).

**Table S1. Bacterial count to assess the bacteriolytic activity of *D. discoideum* extracts.** Bacterial particules were detected on pictures and separated in 3 categories: “debris” (inferior to 0,9 μm^2^), “single bacteria” (between 0,9 and 2 μm^2^) or aggregates (superior to 2 μm^2^) depending on their area.

**Table S2. Full list of proteins with a SS or TMD detected in this study.** A list of all proteins detected with a signal sequence (SS) or transmembrane domain (TMD) in this study is provided. For each protein, the Uniprot number, as well as its gene name, and the number of peptides detected for a given protein (spectrum count) by mass spectrometry in a fraction are indicated.
