# Supplementary material for: Antibacterial effectors in Dictyostelium discoideum: specific activity against different bacterial species
Source: mSphere. 2024 Oct 8;9(10):e00471-24. doi: 10.1128/msphere.00471-24 (PMC11520349; doi:10.1128/msphere.00471-24)
Supplement: Table S1 — Bacterial count to assess the bacteriolytic activity of D. discoideum extracts. [file msphere.00471-24-s0008.docx]

|  | **Kp** | |  | **Ec** | |  | **Pa** | |  | **Sa** | |  | **Bs** | |
| --- | --- | --- | --- | --- | --- | --- | --- | --- | --- | --- | --- | --- | --- | --- |
| **Count** | **Buffer** | **Dd extract** |  | **Buffer** | **Dd extract** |  | **Buffer** | **Dd extract** |  | **Buffer** | **Dd extract** |  | **Buffer** | **Dd extract** |
| **Debris**  **[<0,9 µm^2^]** | 83 | 8966 |  | 30 | 184 |  | 82 | 8591 |  | 6 | 108 |  | 52 | 35 |
| **Single bacteria**  **[0,9;2 µm^2^]** | 9797 | 2439 |  | 69 | 751 |  | 20255 | 1902 |  | 50 | 170 |  | 82 | 140 |
| **Aggregates**  **[>2 µm^2^]** | 1879 | 111 |  | 3273 | 1489 |  | 1275 | 50 |  | 551 | 287 |  | 1539 | 1134 |
|  |  |  |  |  |  |  |  |  |  |  |  |  |  |  |
| **Average size of aggregate (µm^2^)** | 2,54 | 2,83 |  | 5,14 | 4,59 |  | 2,68 | 3,95 |  | 17,24 | 9,97 |  | 6,96 | 4,89 |
| **Total area covered by bacteria (µm^2^)** | 20349 | 3078 |  | 16945 | 7917 |  | 29428 | 2128 |  | 9586 | 3106 |  | 10829 | 5777 |
