# Supplementary material for: Antibacterial effectors in Dictyostelium discoideum: specific activity against different bacterial species
Source: mSphere. 2024 Oct 8;9(10):e00471-24. doi: 10.1128/msphere.00471-24 (PMC11520349; doi:10.1128/msphere.00471-24)
Supplement: Table S2 — Full list of proteins with a SS or TMD detected in this study. [file msphere.00471-24-s0009.docx]

|  |  | **AE resin (mM of NaCl)** | | | | | | | |  | **SEC (mL of elution):** | | | | | | | | |
| --- | --- | --- | --- | --- | --- | --- | --- | --- | --- | --- | --- | --- | --- | --- | --- | --- | --- | --- | --- |
| **Uniprot** | **Gene name** | **50** | **100** | **150** | **200** | **250** | **300** | **350** | **400** |  | **13** | **13,5** | **14** | **14,5** | **15** | **15,5** | **16** | **16,5** | **17** |
| O76856 | ctsD | 95 | 112 | 166 | 90 | 37 | 24 | 7 | 5 |  | 9 | 29 | 37 | 32 | 27 | 10 | 10 | 3 | 4 |
| Q54G77 | tpp1B | 65 | 70 | 84 | 72 | 44 | 32 | 20 | 10 |  | 8 | 25 | 44 | 48 | 39 | 16 | 20 | 14 | 9 |
| Q54TD0 | tpp1F | 18 | 30 | 63 | 82 | 82 | 64 | 21 | 18 |  | 11 | 21 | 19 | 14 | 15 | 7 | 8 | 6 | 7 |
| Q55CF6 | DDB_G0270074 | 19 | 28 | 49 | 57 | 41 | 25 | 6 | 6 |  | 13 | 20 | 17 | 10 | 10 | 0 | 0 | 0 | 0 |
| Q55CF8 | pldX | 10 | 19 | 42 | 43 | 39 | 32 | 16 | 15 |  | 6 | 13 | 19 | 16 | 13 | 6 | 6 | 3 | 0 |
| Q95UC5 | tpp1C | 4 | 12 | 22 | 42 | 39 | 39 | 19 | 11 |  | 5 | 6 | 5 | 0 | 0 | 0 | 0 | 0 | 0 |
| Q55D50 | gaa | 8 | 17 | 41 | 44 | 28 | 19 | 7 | 6 |  | 12 | 8 | 7 | 4 | 2 | 0 | 0 | 0 | 0 |
| Q54CF7 | DDB_G0293014 | 8 | 18 | 28 | 31 | 26 | 20 | 13 | 8 |  | 13 | 16 | 12 | 7 | 5 | 3 | 4 | 3 | 2 |
| Q54J53 | rliF | 9 | 13 | 25 | 33 | 32 | 17 | 9 | 6 |  | 6 | 12 | 7 | 3 | 2 | 0 | 0 | 0 | 0 |
| Q556V7 | DDB_G0273791 | 2 | 4 | 16 | 25 | 27 | 24 | 9 | 7 |  | 11 | 6 | 4 | 3 | 2 | 0 | 0 | 0 | 0 |
| Q55BA2 | bpiC | 6 | 14 | 23 | 28 | 19 | 13 | 6 | 3 |  | 10 | 11 | 8 | 5 | 0 | 0 | 0 | 0 | 0 |
| Q54SA1 | pldZ | 12 | 17 | 34 | 30 | 11 | 6 | 0 | 0 |  | 16 | 14 | 7 | 0 | 0 | 0 | 0 | 0 | 0 |
| Q54ET7 | DDB_G0291344 | 4 | 9 | 27 | 20 | 13 | 6 | 0 | 0 |  | 4 | 9 | 10 | 4 | 4 | 0 | 0 | 0 | 0 |
| Q8T2U3 | DDB_G0275107 | 3 | 6 | 18 | 21 | 12 | 7 | 4 | 2 |  | 7 | 9 | 8 | 5 | 5 | 2 | 0 | 0 | 0 |
| Q54QZ5 | treh | 4 | 8 | 15 | 14 | 8 | 4 | 0 | 0 |  | 2 | 0 | 0 | 0 | 0 | 0 | 0 | 0 | 0 |
| Q54R55 | ctsZ | 11 | 17 | 19 | 18 | 9 | 3 | 0 | 0 |  | 8 | 16 | 16 | 11 | 10 | 4 | 6 | 4 | 0 |
| P54639 | cprD | 6 | 10 | 18 | 14 | 11 | 9 | 4 | 3 |  | 3 | 4 | 4 | 5 | 8 | 0 | 0 | 0 | 0 |
| Q54H23 | DDB_G0289749 | 5 | 10 | 18 | 15 | 12 | 4 | 2 | 0 |  | 6 | 8 | 6 | 0 | 0 | 0 | 0 | 0 | 0 |
| Q54PS7 | plbD | 0 | 11 | 16 | 20 | 10 | 7 | 0 | 0 |  | 5 | 8 | 7 | 4 | 0 | 0 | 0 | 0 | 0 |
| Q54LY3 | badC | 3 | 10 | 15 | 18 | 10 | 9 | 3 | 3 |  | 0 | 0 | 2 | 5 | 7 | 5 | 4 | 3 | 0 |
| Q554H5 | plbF | 0 | 9 | 16 | 19 | 7 | 9 | 0 | 0 |  | 10 | 16 | 14 | 8 | 2 | 0 | 0 | 0 | 0 |
| Q54WD2 | DDB_G0279747 | 4 | 7 | 15 | 14 | 13 | 8 | 0 | 4 |  | 8 | 8 | 7 | 3 | 5 | 0 | 0 | 0 | 0 |
| Q54L19 | uduG | 0 | 8 | 20 | 16 | 9 | 4 | 0 | 0 |  | 0 | 0 | 0 | 0 | 3 | 0 | 0 | 0 | 0 |
| Q54T73 | DDB_G0281967 | 8 | 10 | 21 | 13 | 5 | 0 | 0 | 0 |  | 0 | 3 | 0 | 0 | 0 | 0 | 0 | 0 | 0 |
| Q54QD9 | ctsB | 8 | 9 | 15 | 11 | 7 | 5 | 0 | 0 |  | 0 | 0 | 3 | 4 | 6 | 3 | 2 | 0 | 0 |
| C7FZY2 | DDB_G0295845 | 3 | 9 | 12 | 14 | 10 | 4 | 0 | 0 |  | 0 | 0 | 5 | 7 | 7 | 3 | 0 | 0 | 0 |
| Q54BU3 | DDB_G0293428 | 6 | 9 | 12 | 11 | 8 | 6 | 0 | 0 |  | 2 | 5 | 4 | 3 | 4 | 0 | 0 | 0 | 0 |
| Q54FV5 | dnase2 | 2 | 2 | 7 | 12 | 11 | 9 | 2 | 0 |  | 0 | 0 | 4 | 4 | 4 | 3 | 3 | 0 | 0 |
| Q54GR7 | DDB_G0289949 | 2 | 9 | 14 | 15 | 7 | 5 | 0 | 0 |  | 4 | 6 | 7 | 2 | 3 | 0 | 0 | 0 | 0 |
| Q54K50 | pldY | 4 | 5 | 14 | 11 | 9 | 3 | 0 | 0 |  | 3 | 3 | 0 | 0 | 0 | 0 | 0 | 0 | 0 |
| Q54FV4 | DDB_G0290645 | 4 | 7 | 7 | 8 | 5 | 5 | 0 | 2 |  | 4 | 4 | 3 | 0 | 0 | 0 | 0 | 0 | 0 |
| Q54TR1 | cfaD | 0 | 5 | 5 | 10 | 11 | 8 | 3 | 6 |  | 2 | 8 | 7 | 9 | 8 | 3 | 4 | 3 | 0 |
| Q55FN1 | plbG | 4 | 8 | 12 | 13 | 7 | 4 | 0 | 0 |  | 5 | 6 | 2 | 0 | 0 | 0 | 0 | 0 | 0 |
| Q54VR1 | DDB_G0280187 | 3 | 5 | 18 | 9 | 5 | 3 | 0 | 0 |  | 0 | 4 | 4 | 3 | 4 | 6 | 0 | 0 | 0 |
| Q869Q8 | cpvl | 0 | 0 | 6 | 11 | 11 | 8 | 5 | 5 |  | 2 | 3 | 2 | 0 | 0 | 0 | 0 | 0 | 0 |
| Q54TU4 | DDB_G0281495 | 3 | 5 | 7 | 9 | 8 | 7 | 3 | 0 |  | 7 | 8 | 6 | 4 | 3 | 0 | 0 | 0 | 0 |
| Q55B10 | melA | 6 | 11 | 14 | 7 | 5 | 2 | 0 | 0 |  | 7 | 8 | 5 | 4 | 4 | 0 | 0 | 0 | 0 |
| **Q55G30** | DDB_G0267848 | 3 | 2 | 5 | 12 | 10 | 7 | 0 | 2 |  | 0 | 0 | 3 | 6 | 3 | 4 | 2 | 0 | 0 |
| Q552B4 | badK | 2 | 6 | 10 | 7 | 6 | 0 | 3 | 0 |  | 0 | 0 | 0 | 0 | 3 | 0 | 0 | 0 | 0 |
| Q55FF9 | DDB0189797 | 0 | 4 | 12 | 9 | 7 | 3 | 0 | 0 |  | 4 | 5 | 4 | 4 | 0 | 0 | 0 | 0 | 0 |
| Q556S7 | badI | 5 | 6 | 9 | 7 | 4 | 6 | 0 | 0 |  | 0 | 2 | 4 | 2 | 4 | 2 | 0 | 0 | 0 |
| Q54TS5 | amyA | 15 | 0 | 2 | 5 | 3 | 5 | 5 | 2 |  | 29 | 40 | 31 | 26 | 13 | 7 | 5 | 0 | 0 |
| Q7M438 | ddiA | 7 | 7 | 11 | 9 | 0 | 0 | 0 | 0 |  | 0 | 0 | 0 | 0 | 0 | 0 | 3 | 0 | 0 |
| Q54WE3 | badS (iliP) | 2 | 6 | 13 | 8 | 5 | 0 | 0 | 0 |  | 0 | 0 | 0 | 0 | 2 | 0 | 0 | 0 | 0 |
| Q7KWU6 | badD | 2 | 7 | 9 | 9 | 7 | 0 | 0 | 0 |  | 0 | 0 | 0 | 0 | 3 | 5 | 0 | 0 | 0 |
| Q55BJ6 | plbB | 0 | 0 | 6 | 8 | 6 | 7 | 3 | 2 |  | 5 | 6 | 4 | 2 | 2 | 0 | 0 | 0 | 0 |
| Q550U9 | plbA | 0 | 0 | 6 | 9 | 7 | 4 | 2 | 0 |  | 7 | 13 | 13 | 8 | 7 | 2 | 0 | 0 | 0 |
| Q54ZI6 | plbE | 0 | 3 | 6 | 8 | 6 | 6 | 2 | 0 |  | 10 | 13 | 10 | 3 | 0 | 0 | 0 | 0 | 0 |
| Q54JJ8 | DDB0187731 | 2 | 4 | 10 | 7 | 5 | 4 | 0 | 0 |  | 3 | 4 | 3 | 0 | 0 | 0 | 0 | 0 | 0 |
| Q54HW8 | DDB_G0289171 | 0 | 0 | 5 | 7 | 9 | 5 | 0 | 5 |  | 2 | 4 | 0 | 0 | 0 | 0 | 0 | 0 | 0 |
| Q54EU8 | DDB0183821 | 6 | 6 | 5 | 5 | 2 | 2 | 2 | 0 |  | 0 | 0 | 0 | 0 | 0 | 0 | 2 | 3 | 3 |
| Q54K12 | DDB0187572 | 2 | 3 | 4 | 6 | 6 | 3 | 0 | 0 |  | 2 | 2 | 3 | 2 | 2 | 0 | 0 | 0 | 0 |
| Q54PR9 | cf60 | 0 | 0 | 5 | 6 | 6 | 4 | 2 | 3 |  | 5 | 3 | 2 | 0 | 0 | 0 | 0 | 0 | 0 |
| Q556M2 | DDB0203006 | 5 | 6 | 6 | 6 | 2 | 0 | 0 | 0 |  | 0 | 2 | 0 | 0 | 0 | 0 | 0 | 0 | 0 |
| Q54RL3 | DDB0185347 | 0 | 0 | 8 | 7 | 3 | 3 | 2 | 0 |  | 8 | 9 | 8 | 3 | 2 | 0 | 0 | 0 | 0 |
| Q54BS2 | DDB0191955 | 5 | 6 | 8 | 5 | 3 | 0 | 0 | 0 |  | 2 | 3 | 5 | 4 | 4 | 0 | 0 | 0 | 0 |
| P13723 | hexa1 | 5 | 4 | 4 | 5 | 3 | 2 | 0 | 0 |  | 20 | 23 | 13 | 5 | 8 | 0 | 2 | 0 | 0 |
| Q86IW0 | DDB0217468 | 4 | 5 | 6 | 5 | 3 | 2 | 0 | 0 |  | 0 | 0 | 3 | 4 | 4 | 4 | 2 | 4 | 0 |
| Q54MN6 | DDB0186702 | 2 | 3 | 6 | 6 | 3 | 2 | 0 | 0 |  | 4 | 0 | 0 | 0 | 0 | 0 | 0 | 0 | 0 |
| Q554K2 | DDB_G0274609 | 4 | 6 | 5 | 3 | 4 | 0 | 0 | 0 |  | 2 | 2 | 0 | 0 | 0 | 0 | 0 | 0 | 0 |
| Q54LN4 | gghA | 0 | 0 | 3 | 4 | 4 | 3 | 2 | 4 |  | 4 | 0 | 2 | 0 | 0 | 0 | 0 | 0 | 0 |
| Q54MA4 | DDB0186806 | 0 | 0 | 3 | 3 | 5 | 5 | 2 | 3 |  | 3 | 7 | 9 | 7 | 7 | 2 | 3 | 0 | 0 |
| Q86IK2 | DDB0168871 | 0 | 0 | 2 | 4 | 3 | 4 | 3 | 4 |  | 2 | 2 | 2 | 0 | 0 | 0 | 0 | 0 | 0 |
| Q54I85 | badE | 3 | 2 | 4 | 3 | 3 | 2 | 0 | 0 |  | 0 | 0 | 0 | 0 | 2 | 0 | 0 | 0 | 0 |
| Q54SB4 | DDB0204837 | 3 | 3 | 4 | 3 | 4 | 0 | 0 | 0 |  | 0 | 3 | 6 | 4 | 2 | 0 | 0 | 0 | 0 |
| Q54QF3 | DDB0185734 | 4 | 3 | 3 | 5 | 0 | 0 | 0 | 0 |  | 0 | 0 | 0 | 0 | 2 | 2 | 2 | 0 | 0 |
| Q55GD0 | DDB0189491 | 0 | 3 | 2 | 4 | 3 | 3 | 0 | 0 |  | 0 | 0 | 2 | 0 | 0 | 0 | 0 | 0 | 0 |
| Q54L73 | badQ | 0 | 3 | 3 | 3 | 3 | 3 | 0 | 0 |  | 0 | 0 | 0 | 0 | 2 | 0 | 0 | 0 | 0 |
| Q55DB1 | DDB0190498 | 4 | 3 | 2 | 4 | 3 | 0 | 0 | 0 |  | 0 | 0 | 2 | 0 | 0 | 0 | 0 | 0 | 0 |
| Q7KWW8 | ctbs1 | 2 | 0 | 3 | 4 | 3 | 2 | 0 | 0 |  | 0 | 2 | 2 | 2 | 0 | 0 | 0 | 0 | 0 |
| Q54B18 | DDB0192219 | 0 | 0 | 2 | 5 | 3 | 0 | 2 | 0 |  | 0 | 0 | 0 | 2 | 0 | 0 | 0 | 0 | 0 |
| Q54X38 | badT | 0 | 0 | 2 | 5 | 4 | 3 | 0 | 0 |  | 0 | 2 | 2 | 2 | 0 | 0 | 0 | 0 | 0 |
| Q54G47 | DDB0188874 | 0 | 3 | 5 | 4 | 4 | 0 | 0 | 0 |  | 4 | 3 | 6 | 4 | 3 | 0 | 0 | 0 | 0 |
| Q54LZ4 | badB | 0 | 2 | 3 | 4 | 3 | 0 | 0 | 0 |  | 0 | 2 | 2 | 0 | 0 | 0 | 0 | 0 | 0 |
| Q8MYE7 | DDB0169268 | 0 | 0 | 2 | 0 | 2 | 0 | 2 | 3 |  | 0 | 0 | 2 | 4 | 4 | 3 | 0 | 0 | 0 |
| Q555E5 | DDB0203213 | 0 | 0 | 3 | 5 | 2 | 0 | 0 | 0 |  | 0 | 3 | 6 | 4 | 2 | 0 | 0 | 0 | 0 |
| Q54M12 | DDB0186893 | 0 | 0 | 0 | 2 | 5 | 5 | 0 | 0 |  | 0 | 5 | 3 | 0 | 0 | 0 | 0 | 0 | 0 |
| Q54WM3 | DDB0205841 | 0 | 2 | 2 | 4 | 3 | 0 | 0 | 0 |  | 0 | 2 | 0 | 0 | 0 | 0 | 0 | 0 | 0 |
| Q54GI7 | DDB_G0290139 | 0 | 0 | 0 | 3 | 4 | 0 | 0 | 0 |  | 6 | 3 | 0 | 0 | 0 | 0 | 0 | 0 | 0 |
| Q54C12 | DDB0191818 | 3 | 4 | 0 | 0 | 2 | 0 | 0 | 0 |  | 6 | 6 | 4 | 0 | 0 | 0 | 0 | 0 | 0 |
| Q54IS1 | DDB0187993 | 2 | 0 | 2 | 2 | 2 | 2 | 0 | 0 |  | 0 | 0 | 2 | 0 | 0 | 0 | 0 | 0 | 0 |
| Q54PS3 | DDB0185967 | 0 | 2 | 4 | 2 | 0 | 0 | 0 | 0 |  | 0 | 0 | 0 | 2 | 0 | 0 | 0 | 0 | 0 |
| Q5XM24 | aprA | 0 | 2 | 2 | 4 | 0 | 0 | 0 | 0 |  | 0 | 3 | 2 | 0 | 0 | 0 | 0 | 0 | 0 |
| Q54WG1 | DDB0205980 | 0 | 0 | 2 | 2 | 3 | 0 | 0 | 0 |  | 0 | 0 | 3 | 2 | 0 | 0 | 0 | 0 | 0 |
| Q55F77 | DDB0189876 | 0 | 0 | 0 | 0 | 0 | 0 | 2 | 0 |  | 0 | 0 | 4 | 2 | 0 | 0 | 0 | 0 | 0 |
| Q54DY7 | DDB_G0291912 | 0 | 0 | 0 | 0 | 2 | 0 | 0 | 0 |  | 3 | 4 | 2 | 2 | 2 | 0 | 0 | 0 | 0 |
| Q54BK2 | dcd2A | 0 | 0 | 2 | 0 | 0 | 0 | 0 | 0 |  | 0 | 3 | 0 | 0 | 0 | 0 | 0 | 0 | 0 |
| Q54M57 | DDB0186849 | 0 | 0 | 2 | 3 | 0 | 0 | 0 | 0 |  | 4 | 9 | 5 | 0 | 0 | 0 | 0 | 0 | 0 |
| Q54SC9 | hexa2 | 0 | 0 | 0 | 2 | 0 | 0 | 0 | 0 |  | 2 | 2 | 0 | 0 | 0 | 0 | 0 | 0 | 0 |
| Q23892 | gluA | 2 | 0 | 0 | 0 | 0 | 0 | 0 | 0 |  | 14 | 12 | 8 | 0 | 0 | 0 | 0 | 0 | 0 |
| Q94504 | cprG | 0 | 0 | 12 | 17 | 17 | 17 | 0 | 4 |  | 0 | 0 | 0 | 0 | 0 | 0 | 0 | 0 | 0 |
| Q54RI2 | DDB0185376 | 3 | 6 | 6 | 7 | 8 | 0 | 0 | 6 |  | 0 | 0 | 0 | 0 | 0 | 0 | 0 | 0 | 0 |
| Q54WR3 | DDB0205795 | 0 | 7 | 12 | 7 | 0 | 0 | 0 | 0 |  | 0 | 0 | 0 | 0 | 0 | 0 | 0 | 0 | 0 |
| Q54PT7 | aplN | 6 | 10 | 6 | 5 | 2 | 0 | 0 | 0 |  | 0 | 0 | 0 | 0 | 0 | 0 | 0 | 0 | 0 |
| Q54G58 | badU | 0 | 4 | 2 | 4 | 4 | 3 | 0 | 0 |  | 0 | 0 | 0 | 0 | 0 | 0 | 0 | 0 | 0 |
| Q86LA4 | badA | 0 | 5 | 3 | 5 | 4 | 3 | 0 | 0 |  | 0 | 0 | 0 | 0 | 0 | 0 | 0 | 0 | 0 |
| Q54LG3 | aplB | 4 | 3 | 3 | 4 | 3 | 2 | 0 | 0 |  | 0 | 0 | 0 | 0 | 0 | 0 | 0 | 0 | 0 |
| P54640 | cprE | 5 | 3 | 5 | 3 | 0 | 0 | 0 | 0 |  | 0 | 0 | 0 | 0 | 0 | 0 | 0 | 0 | 0 |
| Q55D04 | badG | 2 | 4 | 4 | 4 | 0 | 0 | 0 | 0 |  | 0 | 0 | 0 | 0 | 0 | 0 | 0 | 0 | 0 |
| Q54HR1 | DDB0188346 | 0 | 2 | 4 | 3 | 4 | 2 | 0 | 0 |  | 0 | 0 | 0 | 0 | 0 | 0 | 0 | 0 | 0 |
| Q553T9 | DDB0202584 | 0 | 2 | 5 | 6 | 0 | 0 | 0 | 0 |  | 0 | 0 | 0 | 0 | 0 | 0 | 0 | 0 | 0 |
| P04988 | cprA | 2 | 0 | 6 | 4 | 3 | 0 | 0 | 0 |  | 0 | 0 | 0 | 0 | 0 | 0 | 0 | 0 | 0 |
| Q54JE8 | DDB_G0288095 | 0 | 0 | 4 | 3 | 2 | 0 | 0 | 2 |  | 0 | 0 | 0 | 0 | 0 | 0 | 0 | 0 | 0 |
| Q54CB7 | DDB0191758 | 0 | 0 | 4 | 4 | 2 | 0 | 0 | 0 |  | 0 | 0 | 0 | 0 | 0 | 0 | 0 | 0 | 0 |
| Q54NI9 | badV | 0 | 0 | 0 | 3 | 3 | 4 | 0 | 0 |  | 0 | 0 | 0 | 0 | 0 | 0 | 0 | 0 | 0 |
| Q54CY6 | badR | 0 | 4 | 4 | 2 | 0 | 0 | 0 | 0 |  | 0 | 0 | 0 | 0 | 0 | 0 | 0 | 0 | 0 |
| Q54L70 | DDB0218865 | 0 | 0 | 4 | 3 | 3 | 0 | 0 | 0 |  | 0 | 0 | 0 | 0 | 0 | 0 | 0 | 0 | 0 |
| Q54TA1 | drkC | 2 | 2 | 2 | 2 | 3 | 0 | 0 | 0 |  | 0 | 0 | 0 | 0 | 0 | 0 | 0 | 0 | 0 |
| Q86HB8 | badW (cfaC) | 3 | 4 | 2 | 2 | 0 | 0 | 0 | 0 |  | 0 | 0 | 0 | 0 | 0 | 0 | 0 | 0 | 0 |
| Q95US4 | gp130 | 0 | 0 | 3 | 4 | 0 | 0 | 0 | 0 |  | 0 | 0 | 0 | 0 | 0 | 0 | 0 | 0 | 0 |
| Q86HR3 | DDB0167672 | 3 | 0 | 2 | 0 | 0 | 0 | 0 | 0 |  | 0 | 0 | 0 | 0 | 0 | 0 | 0 | 0 | 0 |
| Q55GM4 | badF | 0 | 0 | 0 | 0 | 2 | 3 | 2 | 0 |  | 0 | 0 | 0 | 0 | 0 | 0 | 0 | 0 | 0 |
| Q54Z64 | badX (cfaA) | 0 | 0 | 3 | 3 | 0 | 0 | 0 | 0 |  | 0 | 0 | 0 | 0 | 0 | 0 | 0 | 0 | 0 |
| P34115 | GP138A | 0 | 0 | 5 | 4 | 0 | 0 | 0 | 0 |  | 0 | 0 | 0 | 0 | 0 | 0 | 0 | 0 | 0 |
| Q86KC1 | DDB_G0274181 | 0 | 2 | 2 | 2 | 2 | 0 | 0 | 0 |  | 0 | 0 | 0 | 0 | 0 | 0 | 0 | 0 | 0 |
| B0G0Z4 | aplG | 2 | 3 | 2 | 2 | 0 | 0 | 0 | 0 |  | 0 | 0 | 0 | 0 | 0 | 0 | 0 | 0 | 0 |
| Q1ZXF4 | DDB_G0294563 | 0 | 2 | 5 | 2 | 0 | 0 | 0 | 0 |  | 0 | 0 | 0 | 0 | 0 | 0 | 0 | 0 | 0 |
| Q54XV8 | DDB0205537 | 0 | 0 | 2 | 0 | 2 | 2 | 0 | 0 |  | 0 | 0 | 0 | 0 | 0 | 0 | 0 | 0 | 0 |
| Q54C16 | sgmB | 0 | 0 | 2 | 2 | 0 | 0 | 0 | 0 |  | 0 | 0 | 0 | 0 | 0 | 0 | 0 | 0 | 0 |
| Q54XZ8 | badY | 0 | 3 | 0 | 0 | 0 | 0 | 0 | 0 |  | 0 | 0 | 0 | 0 | 0 | 0 | 0 | 0 | 0 |
| Q54CZ8 | DDB0184474 | 0 | 0 | 3 | 2 | 0 | 0 | 0 | 0 |  | 0 | 0 | 0 | 0 | 0 | 0 | 0 | 0 | 0 |
| Q54W28 | badZ | 0 | 0 | 0 | 2 | 0 | 0 | 0 | 0 |  | 0 | 0 | 0 | 0 | 0 | 0 | 0 | 0 | 0 |
| Q54XI0 | DDB0206285 | 0 | 3 | 3 | 0 | 0 | 0 | 0 | 0 |  | 0 | 0 | 0 | 0 | 0 | 0 | 0 | 0 | 0 |
| Q54IE5 | DDB0188120 | 0 | 0 | 3 | 5 | 0 | 0 | 0 | 0 |  | 0 | 0 | 0 | 0 | 0 | 0 | 0 | 0 | 0 |
| Q75JT0 | badH | 0 | 2 | 2 | 2 | 0 | 0 | 0 | 0 |  | 0 | 0 | 0 | 0 | 0 | 0 | 0 | 0 | 0 |
| Q54U18 | DDB0218302 | 0 | 0 | 4 | 0 | 0 | 0 | 0 | 0 |  | 0 | 0 | 0 | 0 | 0 | 0 | 0 | 0 | 0 |
| Q54DI2 | DDB0184285 | 0 | 2 | 2 | 0 | 0 | 0 | 0 | 0 |  | 0 | 0 | 0 | 0 | 0 | 0 | 0 | 0 | 0 |
| Q54H45 | drkB | 0 | 0 | 0 | 3 | 0 | 0 | 0 | 0 |  | 0 | 0 | 0 | 0 | 0 | 0 | 0 | 0 | 0 |
| Q54RM4 | DDB0185336 | 0 | 2 | 2 | 0 | 0 | 0 | 0 | 0 |  | 0 | 0 | 0 | 0 | 0 | 0 | 0 | 0 | 0 |
| Q54CS6 | dcd1B | 0 | 0 | 0 | 2 | 2 | 0 | 0 | 0 |  | 0 | 0 | 0 | 0 | 0 | 0 | 0 | 0 | 0 |
| Q5VJL3 | gdt9 | 0 | 0 | 3 | 0 | 0 | 0 | 0 | 0 |  | 0 | 0 | 0 | 0 | 0 | 0 | 0 | 0 | 0 |
| Q7KWR6 | badL | 0 | 0 | 0 | 3 | 0 | 0 | 0 | 0 |  | 0 | 0 | 0 | 0 | 0 | 0 | 0 | 0 | 0 |
| Q8T293 | ctbs2 | 0 | 0 | 2 | 0 | 0 | 0 | 0 | 0 |  | 0 | 0 | 0 | 0 | 0 | 0 | 0 | 0 | 0 |
| Q54F52 | aplM | 0 | 0 | 0 | 2 | 0 | 0 | 0 | 0 |  | 0 | 0 | 0 | 0 | 0 | 0 | 0 | 0 | 0 |
| Q54VN4 | DDB0230150 | 0 | 0 | 2 | 0 | 0 | 0 | 0 | 0 |  | 0 | 0 | 0 | 0 | 0 | 0 | 0 | 0 | 0 |
| P34098 | manA | 0 | 0 | 0 | 0 | 0 | 0 | 0 | 0 |  | 19 | 19 | 13 | 6 | 5 | 0 | 0 | 0 | 0 |
| Q86B07 | DDB0168980 | 0 | 0 | 0 | 0 | 0 | 0 | 0 | 0 |  | 6 | 10 | 7 | 4 | 3 | 0 | 0 | 0 | 0 |
| Q54TC4 | DDB0204253 | 0 | 0 | 0 | 0 | 0 | 0 | 0 | 0 |  | 3 | 5 | 3 | 0 | 0 | 0 | 0 | 0 | 0 |
| Q54GE1 | glb1 | 0 | 0 | 0 | 0 | 0 | 0 | 0 | 0 |  | 7 | 4 | 2 | 0 | 0 | 0 | 0 | 0 | 0 |
| Q55GC7 | sgmD | 0 | 0 | 0 | 0 | 0 | 0 | 0 | 0 |  | 3 | 3 | 0 | 0 | 0 | 0 | 0 | 0 | 0 |
| Q54VR2 | DDB0206428 | 0 | 0 | 0 | 0 | 0 | 0 | 0 | 0 |  | 0 | 4 | 2 | 0 | 0 | 0 | 0 | 0 | 0 |
| Q54JH2 | DDB0187758 | 0 | 0 | 0 | 0 | 0 | 0 | 0 | 0 |  | 0 | 0 | 0 | 0 | 0 | 3 | 0 | 0 | 0 |
| Q55BZ5 | dcd1A | 0 | 0 | 0 | 0 | 0 | 0 | 0 | 0 |  | 0 | 0 | 2 | 0 | 0 | 0 | 0 | 0 | 0 |
